# Metabolic engineering provides insight into the regulation of thiamin biosynthesis in plants

**DOI:** 10.1093/plphys/kiab198

**Published:** 2021-05-04

**Authors:** Simon Strobbe, Jana Verstraete, Christophe Stove, Dominique Van Der Straeten

**Affiliations:** 1 Laboratory of Functional Plant Biology, Department of Biology, Ghent University, B-9000 Ghent, Belgium; 2 Laboratory of Toxicology, Department of Bioanalysis, Ghent University, B-9000 Ghent, Belgium

## Abstract

Thiamin (or thiamine) is a water-soluble B-vitamin (B1), which is required, in the form of thiamin pyrophosphate, as an essential cofactor in crucial carbon metabolism reactions in all forms of life. To ensure adequate metabolic functioning, humans rely on a sufficient dietary supply of thiamin. Increasing thiamin levels in plants via metabolic engineering is a powerful strategy to alleviate vitamin B1 malnutrition and thus improve global human health. These engineering strategies rely on comprehensive knowledge of plant thiamin metabolism and its regulation. Here, multiple metabolic engineering strategies were examined in the model plant *Arabidopsis thaliana*. This was achieved by constitutive overexpression of the three biosynthesis genes responsible for B1 synthesis, *HMP-P synthase (THIC*), *HET-P synthase* (*THI1*), and *HMP-P kinase/TMP pyrophosphorylase* (*TH1*), either separate or in combination. By monitoring the levels of thiamin, its phosphorylated entities, and its biosynthetic intermediates, we gained insight into the effect of either strategy on thiamin biosynthesis. Moreover, expression analysis of thiamin biosynthesis genes showed the plant’s intriguing ability to respond to alterations in the pathway. Overall, we revealed the necessity to balance the pyrimidine and thiazole branches of thiamin biosynthesis and assessed its biosynthetic intermediates. Furthermore, the accumulation of nonphosphorylated intermediates demonstrated the inefficiency of endogenous thiamin salvage mechanisms. These results serve as guidelines in the development of novel thiamin metabolic engineering strategies.

## Teaser

Metabolic engineering of thiamin (vitamin B1) content emphasizes the need to balance the two branches of B1 biosynthesis and uncovers the importance of B1 salvage to improve engineering strategies.

## Introduction

Thiamin(e) is a water-soluble B-vitamin (B1), which serves as an essential factor in central carbon metabolism in all living organisms (Lonsdale, 2006; Goyer, 2017). There are different chemical states, called vitamers, in which this vitamin naturally occurs, the most predominant being thiamin (nonphosphorylated), thiamin monophosphate (TMP), and thiamin pyrophosphate (TPP), here collectively referred to as B1. TPP serves as a cofactor in multiple crucial enzymatic steps in carbon metabolism, including those catalyzed by pyruvate dehydrogenase, α-keto-glutarate dehydrogenase and transketolase (Goyer, 2010). In doing so, vitamin B1, in the form of TPP, is required in glycolysis, the tricarboxylic acid cycle, nucleotide metabolism, and the synthesis of branched chain amino acids (Adeva-Andany et al., 2017; Strobbe and Van Der Straeten, 2018). Humans, lacking the ability to synthesize this vitamin *de novo*, require adequate supply of B1 vitamins from their diet to ensure normal cellular functioning. However, humans are able to interconvert thiamin and its phosphorylated forms (TMP and TPP; Rao and butterworth, 1995; Nosaka et al., 2001). Therefore, thiamin, TMP, and TPP are able to abate thiamin deficiency in humans (Nabokina et al., 2014), and hence are referred to as B1-vitamers. Insufficient acquisition of B1 vitamers can pose a serious threat to human health, as deficiency can result in neurodegeneration as well as cardiovascular pathologies (Abdou and Hazell, 2015). Unfortunately, thiamin deficiency is a persisting global health problem, affecting many populations in Southeast Asia, as well as the elderly population in the Western world (Hoffman, 2016; Whitfield et al., 2018; Johnson et al., 2019). Several approaches can be followed to reduce the prevalence of thiamin deficiency on a global scale. These approaches include thiamin supplementation and food fortification as well as educational efforts, aimed at achieving dietary diversification of the affected populations (Titcomb and Tanumihardjo, 2019). Alternatively, biofortification, which involves the increase of micronutrient levels in crop plants, can be employed as a cost-effective one-time investment to supply adequate amounts of micronutrients to the populations in need (De Steur et al., 2015; Bouis and Saltzman, 2017). Biofortification can be achieved either by conventional breeding or metabolic engineering using genetic engineering strategies (Strobbe and Van Der Straeten, 2018). Breeding toward higher vitamin content of crops requires high variation in vitamin content of sexually compatible crop germplasm collections (Garg et al., 2018; CAST, 2020; Van Der Straeten et al., 2020). Biofortification via genetic engineering, on the other hand, relies on extensive fundamental knowledge of plant vitamin biosynthesis, salvage, regulation, and impact on general plant physiology, in which pinpointing the factors enabling vitamin accumulation or reduction is pivotal (Strobbe et al., 2018). With regard to these aspects, much remains to be discovered concerning plant thiamin metabolism. Fortunately, all genes participating in *de novo* thiamin biosynthesis in plants have been recently characterized in the model plant Arabidopsis (*Arabidopsis thaliana*).

**Figure 1:**
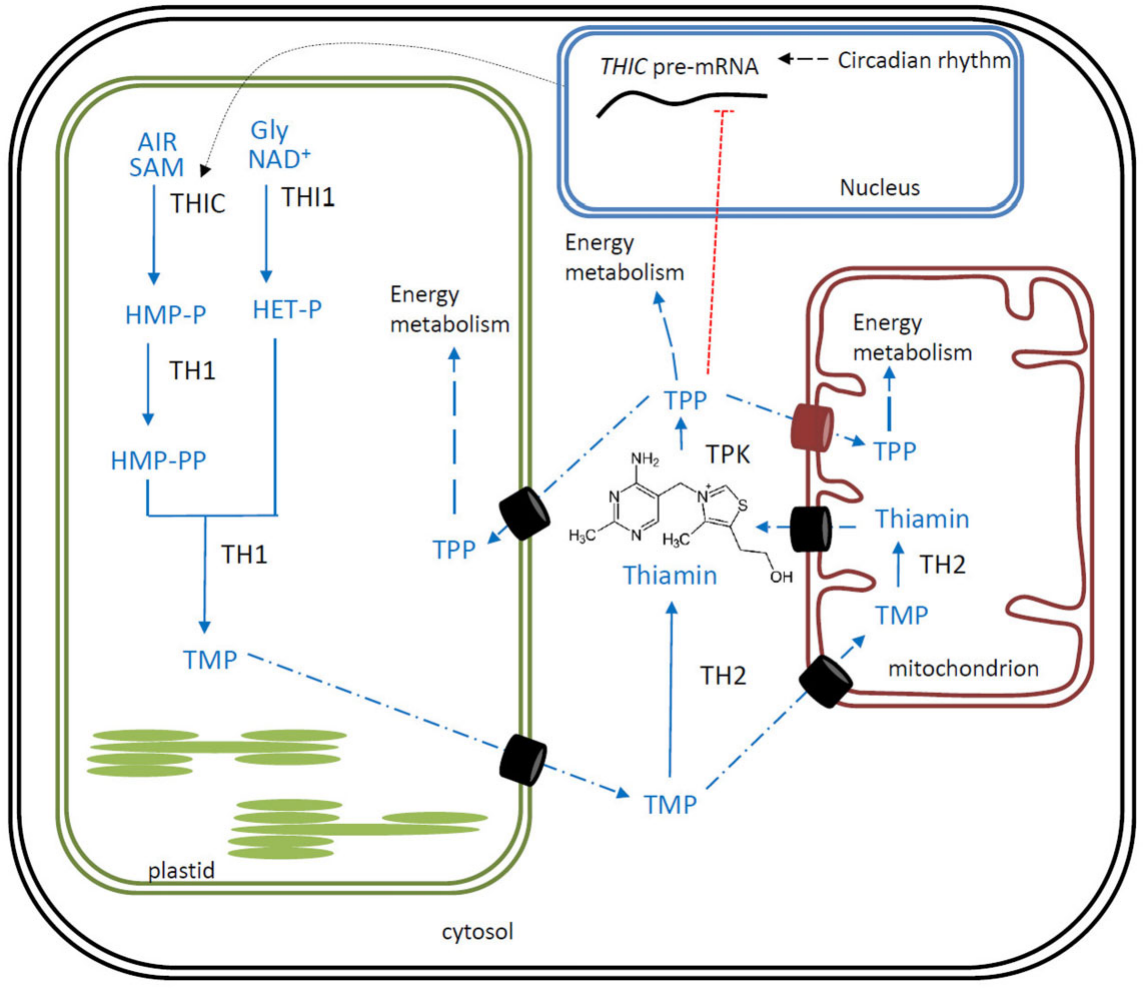
Thiamin biosynthesis in plants. Comprehensive schematic representation of thiamin biosynthesis *in planta* (adapted from Strobbe and Van Der Straeten, 2018; Strobbe et al., 2021). Synthesis of pyrimidine and thiazole moieties as well as their condensation occurs in plastids. Biosynthesis pathway is shown in blue, enzymes in black. Transport across membranes is proposed to be carrier-mediated (barrels), of which the identified mitochondrial TPP carrier is indicated (red barrel; Frelin et al., 2012). The chemical structure of thiamin is depicted, which can be pyrophosphorylated by the action of TPK on its free hydroxyl group. End-product feedback via sensing of TPP levels by the riboswitch residing on the 3′-UTR of *THIC* (pre-mRNA) in the nucleus is shown in red. Products: Gly, glycine; NAD^+^, nicotinamide adenine dinucleotide; Enzymes: THIC, HMP-P synthase; THI1, HET-P synthase; TH1, HMP-P kinase/TMP pyrophosphorylase; TPK; TH2, TMP phosphatase.

In plants, biosynthesis of TPP, the end-product of B1 biosynthesis, is defined by intricate subcellular compartmentalization, as it is found to occur in plastids, cytosol, and mitochondria (Figure 1). Thiamin consists of a pyrimidine moiety linked to a thiazole component, both of which are synthesized in the plastids. The pyrimidine moiety, in the form of 4-amino-2-methyl-5-hydroxymethylpyrimidine phosphate (HMP-P), is created by the action of HMP-P synthase (*THIC, At2g29630*), requiring 5-aminoimidazole ribonucleotide (AIR) and S-adenosylmethionine (SAM) as substrates (Raschke et al., 2007; Kong et al., 2008). The thiazole moiety, 4-methyl-5-β-hydroxyethylthiazole phosphate (HET-P), on the other hand, is generated via the action of HET-P synthase (*THI1*, *At5g54770*; Godoi et al., 2006), which provides the required sulfur from a cysteine residue in the active site (Sun et al., 2019). Hence, the THI1 enzyme undergoes only a single turnover and can therefore be considered suicidal (Garcia et al., 2014). Subsequently, the bifunctional enzyme HMP-P kinase/TMP pyrophosphorylase (*TH1, At1g22940*) phosphorylates HMP-P and condenses the arising product, HMP-pyrophosphate (HMP-PP), with HET-P, giving rise to TMP in the plastids (Ajjawi et al., 2007b). TMP is then metabolized to thiamin via the action of TMP phosphatase (*TH2*, *At5g32470*), which is proposed to be cytosolic (Mimura et al., 2016) as well as mitochondrial (Hsieh et al., 2017). The resulting thiamin can be converted to the active cofactor, TPP, by the action of cytosolic thiamin pyrophosphokinase (TPK), which is encoded by two *TPK* genes in Arabidopsis (*TPK1*, *At1g02880*; *TPK2*, *At2g44750*; Ajjawi et al., 2007a). After the completion of the biosynthesis in the cytosol, TPP can be relocated to the plastids and mitochondria where it is needed for proper metabolic functioning. The subcellular compartmentalization of TPP biosynthesis as well as its requirement in different organelles suggests the existence of intracellular B1 transporters, many of which remain to be characterized (Figure 1), as only the mitochondrial TPP carrier has been identified (Frelin et al., 2012).

The biosynthesis of TPP is known to be tightly controlled, most notably by regulation of the expression of *THIC*. This gene represents an intriguing example of end-product feedback regulation via riboswitch control, a unique mechanism in eukaryotes (Wachter et al., 2007; Bocobza et al., 2013; Srivastava et al., 2018). The riboswitch resides in the three prime untranslated region (3′-UTR) of the *THIC* pre-messenger RNA (mRNA), which, upon binding with TPP, results in the formation of an unstable splice variant. This riboswitch is thought to function as a relief valve to adapt TPP biosynthesis according to specific metabolic requirements (Noordally et al., 2020). Therefore, *THIC* expression can be considered an important regulatory point in plant B1 biosynthesis, further illustrated by the circadian control, mediated by its promoter (Bocobza et al., 2013), as well as its upregulation in certain stress situations (Rapala-Kozik et al., 2012), coinciding with altered levels of B1. On top of the control on *THIC* and its riboswitch mechanism, plants are able to adapt their TPP biosynthesis by control on other biosynthesis genes, including *THI1*, *TH1*, and *TPK* (Rapala-Kozik et al., 2012; Bocobza et al., 2013; Kamarudin et al., 2017). However, the interplay between these components in terms of their regulation, as well as in their role to allow *in planta* accumulation of B1 vitamers and pathway intermediates, remains largely unexplored.

Recently, attention has been drawn toward metabolic engineering of plant thiamin content as a means to enhance nutritional value while improving stress resilience (Dong et al., 2015, 2016; Goyer, 2017; Strobbe and Van Der Straeten, 2018; Fitzpatrick and Chapman, 2020). The reasoning behind this is that enhancement of *in planta* thiamin levels, whether achieved by exogenous application or by metabolic engineering, increases tolerance to biotic (Bahuguna et al., 2012; Dong et al., 2015; Vinchesi et al., 2017; Hamada et al., 2018) and abiotic stresses, such as salt/oxidative stress (Yee et al., 2016; Kaya et al., 2018; El-Shazoly et al., 2019) and drought (Li et al., 2016; Ghafar et al., 2019). The increase of plant thiamin levels is, therefore, highly encouraged, as it would ameliorate global health as well as aid in ensuring sufficient crop yield by tackling stress tolerance. A prerequisite to allow the design and correct implementation of these metabolic engineering strategies involves functional characterization of thiamin biosynthesis genes as well as in depth knowledge on the regulation of plant B1 metabolism. Fine-tuning of metabolic engineering approaches aims to acquire a desired metabolic outcome by engineering a minimal number of genes and limiting undesired metabolic side effects, such as over-accumulation of intermediates (Storozhenko et al., 2007; Farre et al., 2014). Therefore, successful metabolic engineering demands fundamental knowledge beyond functional characterization of the (rate-limiting) biosynthetic enzymes, as in-depth understanding of the different regulatory components controlling biosynthetic flux is essential. In the case of thiamin, tight regulation on biosynthesis and transport has been revealed (Noordally et al., 2020). However, it is not known to what extent the inherent regulation of B1 biosynthesis could hamper metabolic engineering approaches aimed at boosting thiamin levels. Although the necessity to engineer both *THIC* and *THI1* to acquire enhanced thiamin levels in Arabidopsis has been suggested earlier (Bocobza et al., 2013; Pourcel et al., 2013; Dong et al., 2015), the additional benefit obtained by including *TH1* in metabolic engineering approaches remains unclear. Furthermore, nothing is known regarding the potential effect of altered biosynthesis gene expression levels on the accumulation of the pathway intermediates, HMP-P and HET-P for the pyrimidine and thiazole branch, respectively. Fortunately, a novel detection method using liquid chromatography–mass spectrometry (LC–MS/MS; Verstraete et al., 2020), allowing accurate acquisition of metabolite levels for B1 entities and the biosynthetic intermediates in Arabidopsis samples, permits a deeper insight into the B1 biosynthetic flux, potentially indicating additional obstacles in thiamin metabolic engineering.

The aim of this research was to acquire deeper insight into the general aspects of metabolic engineering of thiamin content in plants. This can be achieved by monitoring the expression level of thiamin biosynthesis genes, as well as their associated metabolites in engineered Arabidopsis seedlings. Here, we provide insight into the metabolic changes upon engineering different parts of the thiamin biosynthesis pathway in plants. By analyzing Arabidopsis seedlings constitutively overexpressing one or multiple thiamin biosynthesis genes, we revealed the role of these genes in controlling the flux toward the different B1 vitamers as well as the biosynthetic intermediates. Insights were acquired by monitoring the change in pyrimidine and thiazole intermediates, previously unexplored in this model plant. Moreover, gene expression analysis highlighted routes of regulation of *de novo* thiamin biosynthesis. Accumulation of certain metabolites appears to be tolerated *in planta*, whereas others are subject to a more strict control mechanism. These results aid in acquiring a better understanding of the complex regulation of plant B1 biosynthesis and can serve as a guideline to develop novel B1 metabolic engineering strategies.

## Results

### Generation of transgenic Arabidopsis lines to boost different branches of thiamin biosynthesis

To investigate the importance of the different steps in plant thiamin biosynthesis and the regulation thereof, we enhanced the expression of the respective genes. Examining engineered lines harboring one or multiple thiamin biosynthesis genes under the control of strong promoters, helped to discover the existence of regulatory mechanisms either enhancing or impeding the accumulation of B1 vitamers. These steps include synthesis of the pyrimidine (executed by THIC) and thiazole (executed by THI1) moieties as well as their condensation to form TMP (by TH1). As the main purpose was to acquire information on thiamin metabolism to aid the design of novel biofortification approaches, the biosynthesis downstream of TMP was not engineered, as this compound, together with thiamin and TPP, can be considered as B1 vitamer.

**Figure 2:**
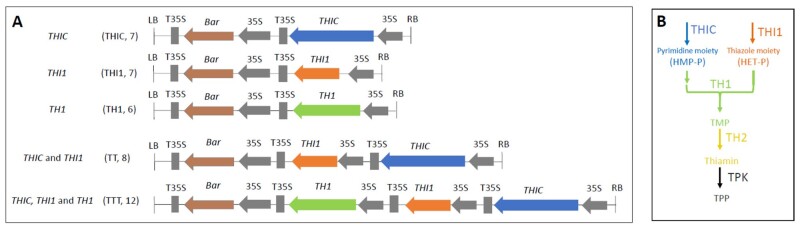
T-DNA inserts used to overexpress one or multiple thiamin biosynthesis genes in Arabidopsis. A, T-DNA inserts in metabolic engineering of the thiamin pathway. Gray arrows and bars represent promoters and terminators, respectively. Colored arrows depict CDS of engineered genes, in which the arrow dimensions of *THIC*, *THI1*, and *TH1* reflect the length of the corresponding CDS. T-DNA constructs were assembled in the binary *PB7wg2.0* plant transformation vector (Karimi et al., 2002). Abbreviations used to refer to the plants harboring the specific construct are depicted between brackets, as well as the number of independent transformation events for which homozygous lines were included. B, Simplified diagram of thiamin biosynthesis. Pyrimidine and thiazole branches are indicated in blue and orange, respectively. TH1 and TMP are indicated in green. TH2 and thiamin are depicted in yellow, while TPK and TPP are indicated in black. LB, left border; RB, right border; T35S, cauliflower mosaic virus 35S terminator; 35S, cauliflower mosaic virus promoter; Bar, marker gene conferring resistance to ammonium glufosinate (Thompson et al., 1987); *THIC*, Arabidopsis HMP-P synthase; *THI1*, Arabidopsis HET-P synthase; *TH1*, Arabidopsis bifunctional HMP-P kinase/TMP pyrophosphorylase; TH2, TMP phosphatase; TPK; TPP.

To achieve this goal, we designed five different T-DNA constructs allowing strong constitutive expression of transgenes driven by the 35S promoter (Figure 2). Three different single-gene vectors were created to overexpress one thiamin biosynthesis gene (either *THIC*, *THI1*, or *TH1*), enabling insight in the role of each gene in altering biosynthetic flux through the B1 biosynthesis pathway. Moreover, we also created multigene constructs harboring two or three biosynthesis genes, providing insight into their potential synergistic functioning. The multigene T-DNA inserts all included the *THIC* transgene. As the endogenous *THIC* gene is strictly controlled by end-product feedback inhibition in its riboswitch (as well as circadian control in its promoter; Bocobza et al., 2013), metabolic engineering approaches in thiamin biofortification will likely all encompass this step. The rationale would be that without *THIC* engineering the plant would still possess a powerful mechanism to control thiamin biosynthesis, being the riboswitch present in the 3′-UTR of the native *THIC*, thereby counteracting such engineering approaches. In addition to *THIC*, *THI1* was chosen as a promising candidate in a two-gene engineering approach. This is based on the knowledge that the thiazole branch of thiamin biosynthesis can become limiting upon adequate supply of pyrimidine, as has been documented in both feeding studies (Pourcel et al., 2013) as well as metabolic engineering (Dong et al., 2015) in Arabidopsis. *TH1* was also included in a trigenic engineering approach given that this gene has the potential to enable adequate condensation of the thiazole and pyrimidine entities toward formation of the B1 vitamer TMP, potentially aiding in reducing the concentration of pathway intermediates. Together, these strategies allow a deeper insight into the metabolic effects of B1 pathway engineering and potentially reveal unknown regulatory mechanisms.

For each T-DNA, 6–12 transgenic lines, resulting from independent plant transformation events, were selected and used to generate homozygous T3 lines.

### Single-gene engineered lines result in high accumulation of intermediates

**Figure 3:**
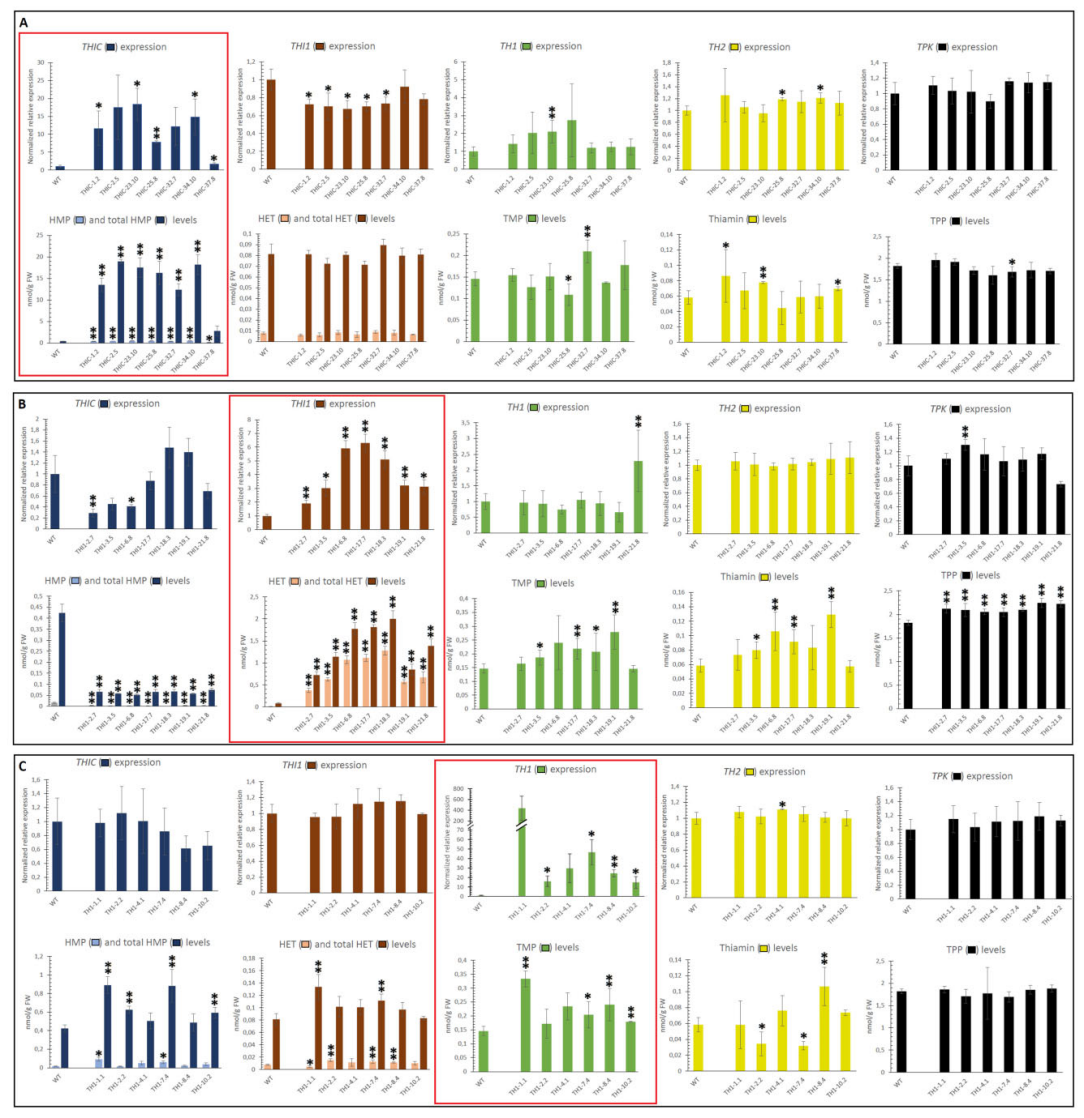
Expression and metabolite analysis of single-gene engineered Arabidopsis lines. Results of *THIC-*engineered lines (A), *THI1*-engineered lines (B), and *TH1*-engineered lines (C) are displayed. All analyses were performed on homozygous T3 lines originating from independent plant transformation events. Values are means (error bars indicate 95% confidence interval) of three independent biological replicates (WT: expression analysis, *N* = 7; metabolites, *N* = 11). Data were obtained from 15-d-old complete seedlings grown on half-strength Murashige and Skoog medium supplemented with 10 g L^−1^ sucrose. Samples were collected in the middle of the light period. Red frames highlight expression of the engineered (overexpression) genes as well as the level of the metabolite arising from the corresponding enzymatic reaction. Statistical difference (in expression or metabolite content) compared to the WT, as obtained by the two-tailed Student’s *t* test, is indicated to be significant by a single asterisk (*P* < 0.05) or very significant by double asterisks (*P* < 0.01). Expression analysis: Relative expression of thiamin biosynthesis genes *THIC*, *THI1*, *TH1, TH2*, and *TPK1/TPK2* is presented (combined expression of endogenous and transgenic), as revealed by RT-qPCR. Values are relative to the mean WT expression which equals 1. Values for ‘*TPK*’ comprise combined expression of *TPK1* and *TPK2* (primers target an amplicon in conserved region, see Supplemental Figure S2B). Specific information regarding primer sequences and conditions can be retrieved from Supplemental Table S1. Metabolite analysis. Quantification of metabolites was performed according to a validated LC–MS/MS method (Verstraete et al., 2020). This methodology includes quantification of HMP, HET, TMP, thiamin, and TPP in both phosphatase-treated and nontreated samples, allowing determination of the sum of nonphosphorylated and phosphorylated metabolites (referred to as ‘total’) as well as nonphosphorylated metabolites, respectively.

Assessing the expression levels of the thiamin biosynthesis genes as well as the quantification of the different metabolites involved in thiamin metabolism provides insight into the effect of the single-gene engineering strategy on the flux through the biosynthesis pathway. Figure 3 shows gene expression and metabolite levels of the *THIC* (Figure 3A), *THI1* (Figure 3B), and *TH1* (Figure 3C) single-gene engineered lines, following the color-code represented in Figure 2B. The gene expression and the associated metabolite content arising from the enzymatic reaction catalyzed by the corresponding protein (e.g. THIC and HMP[-P] in THIC lines) are highlighted with red frames (Figure 3), allowing rapid identification of correlations between gene expression and metabolite levels. Wild-type (WT) Arabidopsis seedlings were found to have a B1 vitamer profile predominantly consisting of TPP, which accounts for ±90% of the B1 pool (TMP, 0.15 nmol g^−1^ fresh weight (FW); thiamin, 0.058 nmol g^−1^ FW; TPP, 1.82 nmol g^−1^ FW). Moreover, WT seedlings were found to have lower molar abundance of the pyrimidine (0.42 nmol g^−1^ FW total HMP) and thiazole (0.08 nmol g^−1^ FW total HET) intermediates, as compared with TPP. The ‘total’ HMP and HET levels were used to indicate the sum of both phosphorylated and nonphosphorylated entities measured by LC–MS/MS after phosphatase treatment (Verstraete et al., 2020).

The THIC lines exhibited elevated expression of the *THIC* transcript, ranging from 1.7-fold (THIC-37.8) to 18-fold (THIC-23.10) above the WT level (Figure 3A, red frame). This coincides with an accumulation of the pyrimidine intermediate HMP(-P) (total HMP) reaching above 18 nmol g^−1^ FW in lines THIC-2.5 and THIC-34.10, which relates to a 40-fold increase as compared to the WT (Figure 3A). Similarly, levels of nonphosphorylated HMP increased approximately 25-fold in the engineered THIC-lines (Figure 3A; Supplemental Figure S1). Line THIC-37.8 was the only exception, showing a less pronounced increase in both phosphorylated and nonphosphorylated HMP metabolites corresponding to its relatively low *THIC* overexpression. Several THIC-lines showed a significant drop in *THI1* expression, which did not result in a decrease in the accumulation of the thiazole intermediate (Figure 3A). Conversely, *TH1* expression appeared to be elevated in the THIC-lines, which was significant in line THIC-23.10 (Figure 3A). This did not, however, have a drastic impact on TMP levels, which were only modestly increased in line THIC-32.7. Overexpression of *THIC* had limited effect on *TH2* expression, although some lines displayed a significant yet modest increase, while a minimal increase in thiamin was also observed. Lastly, overexpression of *THIC*, as well as the accumulation of HMP(-P), did not result in a notable increase of TPP levels (nor *TPK* expression).

The seven engineered THI1-lines examined all displayed a significant increase in *THI1* transcript levels (Figure 3B), corresponding to a remarkable enhancement in both phosphorylated and nonphosphorylated thiazole intermediates. These intermediates increased, as compared to the WT, up to 24- and 160-fold in line THI1-18.3 for total HET and (nonphosphorylated) HET, respectively (Figure 3B; Supplemental Figure S1). As a result, these THI1-lines accumulated the thiazole intermediate predominantly in the form of nonphosphorylated HET. This is in contrast to the WT in which nonphosphorylated HET only accounts for approximately 10% of the total HET pool (WT: 7.74 pmol g^−1^ FW HET; 81.3 pmol g^−1^ FW total HET). Overexpression of *THI1* has an effect on the pyrimidine branch of thiamin biosynthesis as both a decrease in HMP(-P) metabolites as well as *THIC* transcript level was observed in multiple engineered THI1-lines. Moreover, an increased flux toward different B1 metabolites was achieved by *THI1* overexpression, as increased levels of TMP, thiamin, and TPP were observed. The latter explains the reported decrease in *THIC* transcript, as increased TPP levels would lead to the creation of an instable *THIC* transcript via riboswitch functioning in the nucleus (Wachter et al., 2007; Noordally et al., 2020).

The engineered TH1 lines had high accumulation of *TH1* transcript but displayed limited alteration in metabolite composition (Figure 3C). Some of these lines, however, demonstrated a small increase in both pyrimidine (HMP and total HMP) and thiazole (HET and total HET) intermediates. The impact on B1 vitamers was most noticeable on TMP levels for the TH1 lines; line TH1-1.1 showed the highest TMP level of all engineered lines (0.33 nmol g^−1^ FW), more than twice the TMP levels in the WT (0.15 nmol g^−1^ FW).

Overall, these single-gene engineered lines confirmed that overexpression of a single B1 vitamin biosynthesis gene has limited effect on the level of thiamin and its phosphate ester (the B1 pool: TMP, thiamin, TPP; Dong et al., 2015). However, we clearly demonstrated that single-gene intervention can have a massive impact on the accumulation of intermediates in the B1 biosynthesis pathway.

### Multigene engineering of thiamin biosynthesis allows increased flux toward B1 vitamers

As single-gene *THIC* and *THI1* strategies enabled high accumulation of the biosynthetic intermediates HMP(-P) and HET(-P), respectively (Figure 3, A and B), we examined their combined overexpression to increase the biosynthetic flux toward B1 vitamers (TT lines). In a second set of multi-gene engineered lines, we included additional overexpression of *TH1* to further boost condensation of the biosynthetic intermediates (TTT lines).

**Figure 4:**
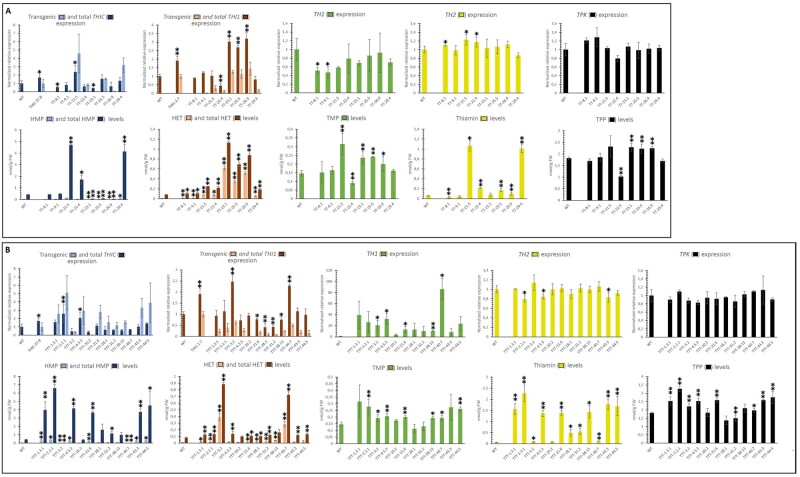
Expression and metabolite analysis of multigene engineered Arabidopsis lines. Results of TT-engineered lines (A) and TTT-engineered lines (B) are displayed. Analyses were performed on homozygous T3 lines originating from independent plant transformation events. Lines TTT-1.3.1, TTT-2.2.1, and TTT-4.5.3 were analyzed in the T4 generation (indicated by their three-digit identifiers). Values are means of three independent biological replicates (error bars indicate 95% confidence interval; WT: expression analysis, *N* = 7; metabolites, *N* = 11). Data were obtained from 15-d-old complete seedlings grown on half-strength Murashige and Skoog medium supplemented with 10 g L^−1^ sucrose. Samples were collected in the middle of the light period. Statistical difference (in expression or metabolite content) compared with the WT, as obtained by the two-tailed Student’s *t* test, is indicated to be significant by a single asterisk (*P* < 0.05) or very significant by double asterisks (*P* < 0.01). Expression analysis: Relative expression of thiamin biosynthesis genes *THIC*, *THI1*, *TH1*, *TH2*, and *TPK* (sum of *TPK1* and *TPK2* transcripts) is presented (combined expression of endogenous and transgenic), as revealed by RT-qPCR. Both total and transgenic expression are presented as ‘relative’ expression. The choice of assigning 1 to WT levels for ‘total’ expression is apparent and in line with earlier representation of the data. For evaluation of the ‘transgenic’ expression, on the other hand, the value of 1 was assigned to the lowest single gene over-expressors (THIC-37.8 and THI1-2.7 for *THIC* and *THI1* expression, respectively). Note that values are expressed relative to the WT and lowest single-gene expressor, respectively; hence, ‘total’ gene expression does not necessarily need to be higher as compared with ‘transgenic’ expression. Transgenic expression of *THIC* and *THI1* was confirmed to be absent in WT samples. Information regarding primer design used to distinguish between total and transgenic expression is provided in Supplemental Figure S2A. Information regarding primer design to assess transcript abundance of both TPK1 and TPK2 is displayed in Supplemental Figure S2B. Information regarding primer sequences and conditions can be retrieved from Supplemental Table S1. Metabolite analysis: Quantification of metabolites was performed according to a validated LC–MS/MS method (Verstraete et al., 2020). This methodology includes quantification of HMP, HET, TMP, thiamin, and TPP in both phosphatase-treated and nontreated samples, allowing determination of the sum of nonphosphorylated and phosphorylated metabolites (referred to as ‘total’) as well as nonphosphorylated metabolites, respectively.

Both sets of multigene engineered lines (TT and TTT) displayed an overall lower increase in total gene expression of *THIC* and *THI1* as compared with the single-gene lines. Therefore, we examined the expression of the transgenic transcripts for *THIC* and *THI1*. Using different primer sets, both total (sum of native and transgenic) and transgenic expression of *THIC* and *THI1* transcripts was investigated by reverse transcription quantitative PCR (RT-qPCR; Supplemental Figure S2). The measured transgenic gene expression is shown relative to the lowest single-gene overexpressor, being THIC-37.8 for *THIC* and THI1-2.7 for *THI1* expression, respectively (Figure 4). This revealed that many lines failed to surmount WT (total) levels of either *THIC* or *THI1* expression, although showing adequate transgenic expression. Indeed, two-gene transgenic lines, TT-25.5 and TT-29.4, as well as several three-gene engineered lines (e.g. TTT-1.3.1, TTT-21.6, and TTT-43.3) displayed transgenic *THIC* expression exceeding the level measured in line THIC-37.8, while having total *THIC* transcript levels not significantly surpassing the WT state (Figure 4). Similar observations were seen for transgenic *THI1* expression in these multigene engineered lines, as multiple lines depicted substantial transgenic *THI1* expression without exceeding the total *THI1* transcript levels of the WT (e.g. TT-21.5, TT-29.4, TTT-1.3.1, and TTT-38.13; Figure 4). Two-gene engineered lines showed a slight decrease in *TH1* expression (Figure 4A), supporting the notion that an additional increase of *TH1* expression could be required, which was successfully achieved in the TTT lines (Figure 4B).

Interestingly, multigene engineered lines showed a tremendous increase in thiamin level, reaching over 1 nmol g^−1^ FW in many lines (TT-21.5, TT-29.4, TTT-1.3.1, TTT-2.2.1, TTT-4.5.3, TTT-21.6, TTT-38.13, TTT-43.3, and TTT-44.5), corresponding to a 17-fold increase. Thiamin levels reached 2.27 nmol g^−1^ FW in line TTT-2.2.1, representing a 39-fold increase over the WT. TMP on the other hand, being the first B1 vitamer formed in *de novo* thiamin biosynthesis, accumulated to a lesser extent in these transgenic lines, with the highest levels reaching just below those in the single-gene engineered line TH1-1.1 (Figures 3C, 4). A substantial increase in TPP level was observed in line TTT-2.2.1, amounting to 3.27 nmol g^−1^ FW, a 1.8-fold increase compared with the WT. Remarkably, line TT-22.4, and to a lesser extent lines TTT-28.1 and TTT-31.2, displayed a decrease in both TPP and TMP levels, while thiamin levels were significantly higher compared to the WT (Figure 4). Line TT-22.4 indeed showed lowered expression of *TPK* and *TH1*, though this was not found to be significant. No clear correlation between *TH2* expression and thiamin accumulation was observed. An evident link between *THI1* expression and HET(-P) accumulation was observed in lines TTT-3.2 and TTT-40.7, which had high *THI1* (trans)gene expression and contained high levels of the thiazole intermediate HET-(P) (Figure 4B;Supplemental Figure S1). However, these lines were found to be depleted in their supply of the pyrimidine intermediate (HMP, HMP-P, and HMP-PP). A similar observation was made for two-gene engineered lines TT-23.1, TT-25.5, and TT-26.9 (Figure 4A;Supplemental Figure S1).

In multigene engineered lines, (transgenic) *THIC* expression appeared to be an important factor determining the accumulation of thiamin, as high (transgenic) *THIC* expression coincided with high thiamin accumulation in many lines (e.g. TT-21.5, TT-29.4, TTT-1.3.1, TTT-2.2.1). The high thiamin levels measured in TT lines showed that accumulation of B1 vitamers is possible without engineering of *TH1*, although thiamin accumulation was more frequently observed and reached higher levels in TTT lines.

**Figure 5:**
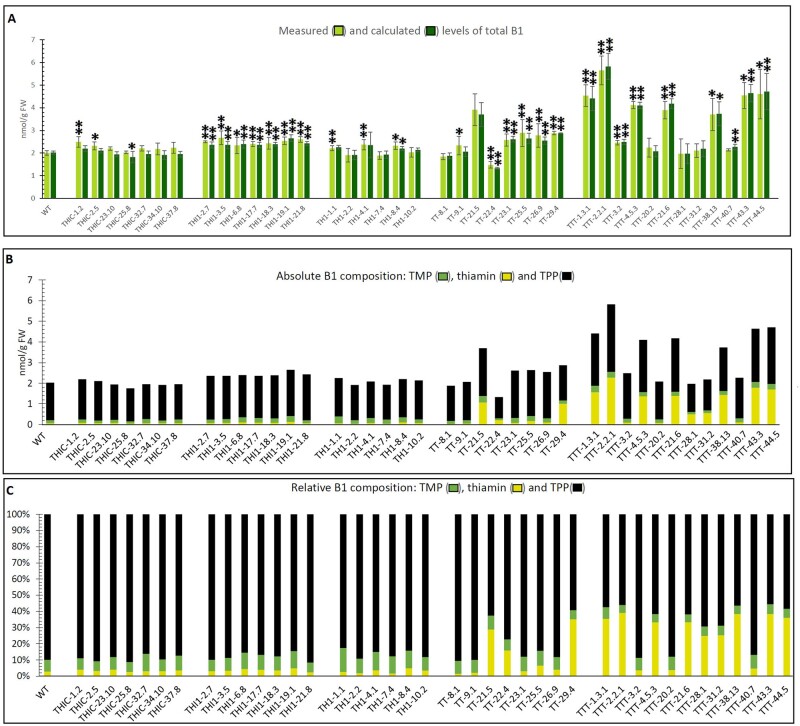
Levels of total B1 (TMP, thiamin, and TPP) in transgenic Arabidopsis seedlings. Analyses were performed on homozygous T3 lines originating from independent plant transformation events. Lines TTT-1.3.1, TTT-2.2.1, and TTT-4.5.3 were analyzed in the T4 generation (indicated by their three-digit identifiers). Data were obtained from 15-d-old complete seedlings grown on half-strength Murashige and Skoog medium supplemented with 10 g L^−1^ sucrose. Samples were collected in the middle of the light period. Quantification of metabolites was performed according to a validated LC–MS/MS method (Verstraete et al., 2020). A, Total B1, being the sum of TMP, thiamin, and TPP, acquired either via direct measurement of thiamin in phosphatase-treated samples (measured), or by summation of TMP, thiamin, and TPP levels detected in nonphosphatase-treated samples (calculated). Values are means of three independent biological replicates (error bars indicate 95% confidence interval; WT: *N* = 11). Statistical difference compared to the WT, as obtained by the two-tailed Student’s *t* test, is indicated to be significant by (*P < 0.05) or very significant by (**P < 0.01). B, The contribution of each B1 vitamer (TMP, thiamin, and TPP) to the calculated total pool of B1 is shown, as measured by LC–MS/MS. C, Relative contribution of each vitamer to the total B1 pool of the engineered Arabidopsis lines (expressed as a percentage of the total).

As the engineering approaches appeared to have severe effects on the accumulation of different metabolites in thiamin metabolism, the impact on total B1 content should be assessed. This is indeed an important parameter, particularly in light of potential translation of the results toward biofortification of food crops. Quantification of total B1 (sum of TMP, thiamin, and TPP) can be done via direct measurement of thiamin in phosphatase-treated samples (Verstraete et al., 2020). Alternatively, the molar concentration of thiamin, TMP, and TPP can be added up to obtain the total B1 present. The observation that the overall measured and calculated (TMP + thiamin + TPP) levels of total B1 in the different lines coincide (Figure 5A) indicates that the presence of thiamin triphosphate is a negligible fraction of total B1 (Makarchikov et al., 2003; Bettendorff et al., 2014; Hofmann et al., 2020).

The largest increases in total B1 content were measured in the TTT lines, the highest being TTT-2.2.1, which exhibited a total B1 content of 5.82 nmol g^−1^ FW, almost three-fold higher than the WT (2.02 nmol g^−1^ FW; Figure 5A). When considering the single-gene engineering approaches, *THI1*-overexpression appeared to be the most successful to increase total B1 levels, achieving a 1.3-fold increase in total B1. In these *THI1* lines, increases in B1 were mainly due to modest augmentation of TPP levels (Figure 5, B and C). The situation was different in multigene engineered lines, where the B1 composition was severely altered in favor of thiamin, as this vitamer represented 35%–40% of the B1 pool in lines TT-29.4, TTT-1.3.1, TTT-2.2.1, TTT-38.13, TTT43.3, and TTT-44.5 (Figure 5C). In WT seedlings, thiamin only encompassed 10% of the B1. The higher relative abundance of the thiamin vitamer, previously documented in engineered Arabidopsis (Dong et al., 2015) and rice (*Oryza sativa*) leaves (Dong et al., 2016), was confirmed in this study. Interestingly, our analysis also demonstrated that the altered B1 profile, reflected in a higher relative abundance of thiamin, can also occur without an increase in total B1 content. Indeed, lines TTT-28.1 and TTT-31.2 exhibited no significant alteration in B1 content (Figure 5, A and B), while the vitamer profile changed as the relative abundance of TPP decreased in favor of thiamin (Figure 5C).

## Discussion

### Natural balance in B1 biosynthesis

The bioactive cofactor TPP, considered the end-product of thiamin biosynthesis, is by far the most abundant form of B1 in Arabidopsis seedlings, confirming what had been observed previously in Arabidopsis tissues (Bocobza et al., 2013; Dong et al., 2015). Furthermore, the molar occurrence of this B1 entity greatly outweighs the observed content of the B1 biosynthesis intermediates pyrimidine and thiazole (Supplemental Figure S3). Taken together, these observations indicate that the natural state of these tissues is adequately capable to steer B1 biosynthesis toward TPP without high accumulation of intermediates. However, it should be noted that the level of the pyrimidine intermediate is substantially higher as compared to that of the thiazole intermediate. The latter might be a result of the high metabolic burden of HET-P production by the suicidal enzyme THI1 (Sun et al., 2019). The observation that the intermediates predominantly occur in their phosphorylated form in the WT further highlights their metabolic role to be readily consumed in B1 biosynthesis. This B1 profile is indicative for an active (*de novo* biosynthesis) metabolism and is in great contrast with what has been reported in seeds of food crops, in which B1 predominantly occurs as thiamin, serving as a stable storage form (Shimizu et al., 1990; Golda et al., 2004; Strobbe et al., 2021).

### Pyrimidine and thiazole intermediates can accumulate to high levels

The engineered lines overexpressing *THIC* and/or *THI1* clearly demonstrate that the pyrimidine as well as the thiazole intermediate can accumulate upon stimulation of the corresponding enzymatic activity. A strong correlation between (trans)gene expression and accumulation of the direct downstream metabolite (e.g. HET-P in *THI1*-overexpressing lines) was witnessed in single-gene engineered lines (Supplemental Figure S4; Figure 3, A and B [red frames]). This observation indicates that the content of these metabolites (HMP(-P) and HET(-P)) is almost exclusively determined by the expression of the genes corresponding to the enzymes carrying out their biosynthesis. Hence, the intermediate metabolites are flexible in a way that metabolic engineering allows their strong accumulation.

Most notably, the pyrimidine intermediate HMP-P(P) reached very high levels in *THIC-*overexpressing lines, exceeding 15 nmol g^−1^ FW in multiple lines (Figure 3A). This accumulation, together with the limited increase in B1 entities, confirms the presence of an additional metabolic limitation, thiazole supply, hampering conversion to B1 (Pourcel et al., 2013; Dong et al., 2015). However, such molar quantities of the pyrimidine intermediate would, if able to undergo downstream biosynthesis reactions, allow an increase in total B1 more than eight-fold compared with the WT. This shows that only a limited increase in *THIC* expression is needed to provide sufficient molar quantities of the pyrimidine intermediate. The increased *THIC* expression and coinciding elevation of pyrimidine intermediates, although not causing an aberrant phenotype when grown in vitro, induced abnormal growth when grown on soil (Supplemental Figures S5 and S6), as has been previously observed (Bocobza et al., 2013; Dong et al., 2015; Yin et al., 2019). Although this altered phenotype had been ascribed to increased TPP production, these results indicate that accumulation of pyrimidine is at least partially responsible for the witnessed phenotypic aberrations, as some of its associated breakdown products are known to be toxic (Zallot et al., 2014). This emphasizes the need to balance pyrimidine production according to the required augmentation of B1 vitamers (avoiding over-production), as well as to ensure sufficient detoxification of arising toxic metabolites (e.g. via HMP salvage by *At3g16990*; Zallot et al., 2014).

The thiazole intermediate, on the other hand, accumulated to much lower levels in the engineered lines compared to the aforementioned high levels of the pyrimidine intermediate. The highest level of total thiazole, measured in line THI1-18.3, only reached 2 nmol g^−1^ FW (Figure 3B), matching the molar quantities of total B1 (Figure 5A). This is almost an order of magnitude lower than the accumulated pyrimidine intermediate witnessed in THIC-engineered lines. It demonstrates that engineering of *THI1* expression, although clearly correlated with the observed increase in thiazole (Supplemental Figure S4), is less powerful in ensuring high production of the intermediate as compared with the pyrimidine accumulation upon *THIC* overexpression. This indicates that insufficient acquisition of the thiazole intermediate, HET-P, hampers higher accumulation of B1 vitamers. This could be attributed to the suicidal nature of the THI1 enzyme, known to consume a substantial portion of the plant’s maintenance energy (Hanson et al., 2018; Sun et al., 2019). Metabolic engineering for higher B1 therefore requires adequate coordination of thiazole and pyrimidine production to allow thiamin accumulation and limit the build-up of intermediates (Supplemental Figure S4).

The high accumulation of nonphosphorylated HET in the *THI1*-overexpressing lines (both single and multigene engineered; Supplemental Figures S1B, 3B and 4A) reveals an opportunity to strengthen the thiazole branch. Nonphosphorylated HET originates from breakdown of B1 vitamers as well as HET-P (Yazdani et al., 2013). The endogenous thiazole salvage mechanism in these lines, consisting of a HET kinase enzyme (corresponding to *At3g24030*), is insufficient to eliminate the generated pool of nonphosphorylated HET. Additional engineering of this step will likely aid in strengthening the thiazole branch by ensuring increased supply of the intermediate HET-P, which can be readily condensed to form B1 vitamers. Such engineering strategy would need to take subcellular localization of both the salvaging enzymes (HET kinase) and the accumulating breakdown product, HET, into consideration. The HET salvaging enzyme, HET kinase, is proposed to be cytosolic, hence requiring a plastidial HET-P transporter to replenish HET-P stock in the plastids (Yazdani et al., 2013). Such a yet-to-be characterized HET-P transporter would be the thiazole salvaging equivalent of the recently characterized HMP transporter *nucleobase cation symporter 1* (*NCS1*, *At5g03555*) which recycles the pyrimidine moiety by mediating its transport from the cytosol to the plastids (Beaudoin et al., 2018). This raises the question whether the supply of intermediates, particularly HET-P, accumulating in the engineered lines, is adequate in plastids (either through synthesis or transporter activity following salvage). Surely, the observed impact of accumulating HET(-P) coinciding with an increase in downstream metabolites (B1-vitamers; Figures 3B and 4), shows that the arising thiazole is indeed utilized in downstream thiamin biosynthesis, thus at least partially present in the plastids. However, it cannot be excluded that inadequate subcellular trafficking is limiting the complete biosynthetic utilization of the intermediates.

### Transcriptional regulation can counteract engineering approaches

Transcriptional analysis of the engineered lines revealed the ability of the plant’s metabolism to respond to the modifications in B1 biosynthesis. In general, these transcriptional reactions involved a counteraction of the engineering approach. This was the case in single-gene engineered lines, where *THIC* overexpression led to a drop in *THI1* expression (Figure 3A). Reciprocally, *THI1* overexpression caused a significant drop in *THIC* expression in multiple lines (Figure 3B). The molecular mechanism guiding the former remains to be characterized, while the latter is attributed to the riboswitch functioning (Wachter et al., 2007; Bocobza et al., 2013), which was activated by higher TPP levels witnessed in these lines.

The ability of plant metabolism to counteract the engineering approach is clearly visible in the multigene engineered lines, in which the transgenic expression of *THIC* and *THI1* was counterbalanced by altered endogenous gene expression. Indeed, many lines depicted promising transgenic expression of both *THIC* and *THI1*, while total (both transgenic and endogenous) expression of *THIC* and *THI1* was found to resemble or even drop below that of the WT, respectively (Figure 4). Therefore, it can be concluded that the plant harbors a powerful regulatory mechanism, able to tune down the expression of the native genes. This transcriptional regulatory mechanism appeared to be confined to *THIC* and *THI1*, although three TTT lines displayed a significant decrease in *TH2* expression, indicative of a similar regulation. This transcriptional flexibility of thiamin metabolism to respond to biosynthetic alterations was demonstrated in the *th2* mutant, which showed upregulation of *THIC*, *THI1*, *TH1*, and *TPK* (Hsieh et al., 2017). Here, the engineered lines illustrate how the endogenous regulatory mechanism is also capable of correcting thiamin metabolism in the case of biosynthesis over-activation.

### Rate limiting steps of B1 biosynthesis

The increase in B1 content in single-gene engineered lines provided insight into the endogenous equilibrium state of thiamin biosynthesis in Arabidopsis. The observation that overexpression of *THI1* had a substantially bigger impact on increasing B1-vitamers as compared with stimulation of *THIC* or *TH1* (Figure 3), reveals that THI1 activity is controlling the flux through the B1 biosynthesis pathway in these conditions. In doing so, the metabolism aligns THI1 activity with B1 biosynthesis requirements to prevent an excess of this energetically expensive suicidal enzyme (Hanson et al., 2018; Sun et al., 2019; Joshi et al., 2020). The high-energy cost was also reflected in the high relative abundance of the *THI1* transcript (Supplemental Figure S7). This functionality as a rate-limiting control point could be an evolutionary explanation for the suicidal nature of this THI1 enzyme, as plant-specific catalytic (nonsuicide) THI1 enzymes have been discovered as well (Joshi et al., 2020). Furthermore, THIC is known to be a control point in thiamin biosynthesis, as its manipulation can result in increased B1 content (Kong et al., 2008; Bocobza et al., 2013; Dong et al., 2015). Here, *THIC* overexpression, although able to raise the B1-vitamers TMP and thiamin, was ineffective in augmenting the levels of TPP (Figure 3A). This confirms the role of the riboswitch, residing in the 3′-UTR of *THIC*, as a relief valve to control thiamin homeostasis (Wachter et al., 2007; Noordally et al., 2020). In the case of lowered metabolic need or inadequate functioning of another control point, such as THI1, the riboswitch in the *THIC* transcript can intervene and shut off the pyrimidine branch of B1 biosynthesis; the latter was witnessed in THI1-engineered lines (strong decrease in pyrimidine levels; Figure 3B). The ability of sole overexpression of *THIC*, deprived of its riboswitch, to augment B1 levels might therefore pertain to certain conditions. The observation that Arabidopsis lines, deprived of proper riboswitch functioning, displayed aberrant phenotypes (Bocobza et al., 2013; which was also witnessed in *THIC*-overexpressing lines in this study [Supplemental Figure S5]), further indicates the shortcomings of engineering strategies solely focusing on *THIC*. This likely impacts the required balancing of the two different branches of B1 biosynthesis. The use of multiple enzymes operating at a rate-limiting pace, thereby serving as control points to determine metabolic (biosynthetic) flux, makes sense given the great requirement of the cell to fine-tune thiamin metabolism (Bocobza et al., 2013; Fitzpatrick and Chapman, 2020; Noordally et al., 2020; Rosado-Souza et al., 2020).

Overexpression of *TH1*, on the other hand, displayed limited effect on B1 vitamer accumulation, being mostly confined to an increase in the direct downstream product TMP (Figure 3C). An increased content of pyrimidine and thiazole intermediates observed in these lines could hint to the existence of a positive feedback loop. However, this observation is likely linked to an increase of biosynthetically inactive intermediate compounds resulting from B1 breakdown, considering the higher levels of nonphosphorylated intermediates measured in these lines (Figure 3C;Supplemental Figure S1). Analysis of the engineered lines indicated that TH1 activity is not limiting B1 accumulation. However, upon stimulation of THIC and THI1, an additional increase in TH1 activity aids in augmenting thiamin content, supported by the higher levels of thiamin as well as higher incidence of thiamin accumulation in TTT lines (compared with TT lines; Figure 4). It should be noted that the transcript abundance of *TH1* in WT Arabidopsis was found to be roughly 100 times lower as compared to that of *THIC*, *TH2*, and *TPK* (Supplemental Figure S7), and almost 7,000 times lower compared with *THI1*, showing that the natural state of thiamin biosynthesis only requires very low *TH1* expression. The lower relative abundance of *TH1* transcripts in the WT state also explains the higher relative increase in *TH1* expression in transgenic lines (Figure 3C). Indeed, *THI1*, having very high transcript abundance in the WT state (Supplemental Figure S7), showed a lower relative increase upon its overexpression.

The engineered lines revealed that TMP phosphatase activity, executed by TH2, is not limiting the metabolic flux toward thiamin and TPP. This can be deduced from the high thiamin accumulation in multigene engineered lines. Indeed, multiple lines (TTT-2.2.1, TTT-4.5.3, and TTT-43.3) had high accumulation of thiamin, the product originating from TH2 functioning (Figure 1), even with a witnessed decrease in *TH2* expression (Figure 4B). This shows that native TH2 functioning is able to handle the increased flux toward thiamin, providing high conversion of TMP to thiamin, thereby only allowing limited accumulation of TMP. Similar to *TH2*, endogenous *TPK* expression appears sufficient to allow modest increases in TPP. It cannot be excluded that overexpression of *TPK* would lead to higher TPP levels. However, overexpression of *TPK* did not result in higher B1 levels in rice leaves (Dong et al., 2016).

### Potential improvement in thiamin metabolic engineering

The engineering approaches in the model plant Arabidopsis revealed some general aspects to be taken into consideration upon conducting metabolic engineering aimed at acquiring increased levels of B1 vitamers. Balancing the production of both thiazole and pyrimidine is required to enable adequate coupling toward B1 vitamins, thereby limiting the accumulation of intermediates, which would pose an energetic burden on metabolism (Hanson et al., 2018; Sun et al., 2019). Efficient usage of biosynthetic intermediates requires stimulation of salvage/detoxification reactions to limit the buildup of compounds unrelated to the biosynthesis. Subcellular localization and metabolic rhythms in the accumulation of different B1 vitamers are also important to consider, as they can be a determinant in biosynthetic feedback control. This notion has been well documented for TPP, known to be imported into nuclei and to play a major role in end-product feedback through riboswitch binding (Bocobza et al., 2013). In addition, it was recently demonstrated that TPP is subject to a circadian clock-independent oscillation in function of light-dark cycles (Noordally et al., 2020). Therefore, it is tempting to speculate whether engineering toward TMP or thiamin is favorable. Similarly, B1 engineering toward specific subcellular compartments to diminish biosynthetic feedback could prove to be beneficial. In this regard, identification of subcellular B1 transporters is pivotal. From a metabolic engineering perspective, a vacuolar thiamin importer would be valuable, as the vacuole could, given its higher acidity, assure adequate stability and likely protects B1 metabolism from feedback on biosynthesis. As to the thiazole branch of B1 biosynthesis, replacing the suicidal THI1 enzyme by a nonsuicidal equivalent would represent a great leap forward in metabolic engineering of B1 (Minhas et al., 2018; Sun et al., 2019), as it would likely result in higher production of the thiazole intermediate, requiring a lower metabolic cost, while improving the energy efficiency of the plant (Hanson et al., 2018; Amthor et al., 2019; Joshi et al., 2020). Such catalytic (i.e. nonsuicidal, able to undergo multiple turnovers) THI1 enzymes have been examined in cereal crops including oat (*Avena sativa*), wheat (*Triticum aestivum*), and barley (*Hordeum vulgare*), and are believed to be present in other grasses (Joshi et al., 2020). Given the potential advantage of nonsuicidal THI1 variants, this enzyme is an ideal target in directed evolution studies (Hanson et al., 2018). In this regard, a system suitable for continuous directed enzyme evolution tested on the nonsuicidal *Thermovibrio ammonificans* THI1 appears promising to obtain higher catalytic efficiency in future plant engineering approaches (Garcia-Garcia et al., 2020). Lastly, additional stimulation of the condensation of the intermediates, executed by TH1, appears to be beneficial to reach the highest levels of thiamin (and thus total B1).

### Extrapolation to other plant species and tissues

It is important to consider to what extent the observations made in Arabidopsis seedlings reflect the situation in other plant species and in different tissues. This is of particular interest given the potential of the acquired knowledge in guiding metabolic engineering strategies aimed at ameliorating the nutritional value as well as stress resilience of crop plants. Arabidopsis remains a useful reference plant to gain fundamental knowledge into plant metabolism, expected to be fairly conserved among many species, including agronomically relevant crops. As discussed above, the tissues examined here are active tissues, performing continuous *de novo* thiamin biosynthesis. Therefore, the observations concerning the accumulation of metabolites, the necessity of balancing both intermediate branches as well as their salvage, and the overall transcriptional control, are proposed to, at least partially, hold true in similar tissues. One can reasonably state that these will include green leafy vegetables, such as kale (*Brassica oleracea*), spinach (*Spinacia oleracea*), and lettuce (*Lactuca sativa*). On the other hand, it is logically predictable that balancing of intermediates will be a universally applicable necessary condition in different plant tissues. Similarly, the limitation that inadequate salvage of (nonphosphorylated) intermediates poses to the flux in the pathway likely holds true in a range of different tissues. Indeed, inadequate salvage of intermediates was proposed to limit thiamin accumulation in rice endosperm (Goyer, 2017; Strobbe et al., 2021), which is a target tissue in biofortification efforts of grain crops and is considered less metabolically active tissue. The observed sufficiency of endogenous TH2 to allow adequate thiamin accumulation was also reported in rice leaves (Dong et al., 2016) and endosperm (Strobbe et al., 2021). Furthermore, in rice endosperm, precursors for pyrimidine biosynthesis upstream of THIC, such as SAM and AIR, are pinpointed as potential limiting factors of thiamin biosynthesis (Goyer, 2017; Strobbe et al., 2021). This is in strong contrast to what was observed in Arabidopsis, where *THIC* overexpression allowed a remarkable increase in pyrimidine accumulation, proving ample supply of biosynthetic precursors. Indeed the accumulation of pyrimidine and thiazole intermediates in Arabidopsis (Figure 3, A and B) largely exceeds the modest accumulation of intermediates observed in rice endosperm (Strobbe et al., 2021). It can be concluded that comprehensive analysis of (engineered) Arabidopsis seedlings provides novel fundamental insights in general thiamin metabolism, although some observations will prove to be tissue-specific or related to environmental conditions.

### Toward a better understanding of thiamin metabolism

As multiple, primarily beneficial effects on plant growth and development have been attributed to thiamin, there is a big incentive to elucidate the role of thiamin in plant physiology. These effects include increased tolerance to biotic (Bahuguna et al., 2012; Vinchesi et al., 2017; Hamada et al., 2018; Sathiyabama et al., 2019) and abiotic stresses such as salt/oxidative (Yee et al., 2016; Kaya et al., 2018; El-Shazoly et al., 2019), heavy metal (Sanjari et al., 2019; Kaya and Aslan, 2020), and drought stress (Li et al., 2016; Ghafar et al., 2019; Jabeen et al., 2020). The recently developed LC–MS/MS methodology to quantify the different thiamin vitamers as well as its biosynthesis intermediates (Verstraete et al., 2020), together with engineered lines exhibiting altered B1 vitamer and/or biosynthesis intermediate content, could aid in pinpointing the exact metabolite provoking a given physiological effect. Indeed, several lines described here, with high thiamin content together with lowered TPP (and TMP) levels (Figure 4; lines TT-22.4, TTT-28.1, and TTT-31.2), could be examined to elucidate which vitamer is causing the physiological effect ascribed to B1.

## Conclusions

Analysis of the engineered lines showed that metabolic engineering of thiamin content results in an exceptional accumulation of its biosynthetic intermediates in Arabidopsis seedlings, though not leading to a spectacular thiamin accumulation. Measurement of pyrimidine and thiazole levels in the transgenic lines further revealed the necessity of balancing the two branches of thiamin biosynthesis to obtain the B1 enhancement required to substantially alleviate thiamin deficiency. This notion is further supported by the indicated harmful impact of high pyrimidine accumulation as well as the metabolic burden of thiazole production. The chemical state (nonphosphorylated) in which the intermediates were observed in the engineered lines emphasizes the necessity of implementing salvage in thiamin metabolic engineering designs. Therefore, it is strongly advised to incorporate assessment of intermediate accumulation as a pivotal part of both the design as well as *post hoc* evaluation of thiamin metabolic engineering strategies. Furthermore, comprehensive assessment of thiamin biosynthesis gene expression as well as vitamer profiling showed that both TH2 and TPK functioning are not limiting B1 accumulation. This suggests that biofortification approaches would therefore not benefit their implementation.

Taken together, these findings allow an unprecedented deeper insight into thiamin metabolism, particularly concerning the effects of accumulation of biosynthesis intermediates, and aid in steering future metabolic engineering approaches.

## Materials and methods

### Molecular cloning and construct design

*Arabidopsis thaliana* cDNA was used to amplify the coding sequences (CDS) of *THIC*, *THI1*, and *TH1* genes. These were cloned into pDONR221 (BP) using the Gateway cloning system (primers depicted in Supplemental Table S1). These were subsequently transferred (LR) into the pre-existing binary plant transformation vector *PB7WG2.0* (Karimi et al., 2002) harboring a 35S promoter and T35S terminator as well as an ammonium glufosinate resistance selection cassette. The different transcriptional units were assembled by digesting the single-gene vector (*PB7-THIC*) with *Spe*I and *Sma*I (liberating the 35S-*THIC*-T35s cassette), followed by blunting reaction (CloneJET PCR Cloning Kit, ThermoFisher Scientific) and blunt-end ligation into the *Sma*I-digested subsequent binary vector (PB7-THI1), yielding the transformation vector containing both transcriptional units (PB7-*THIC*-*THI1*). Similarly, the *35S-TH1-T35S* cassette was cloned into the *PB7-THIC-THI1* vector to create the *PB7-THIC-THI1-TH1* vector. This resulted in the creation of binary plant transformation vectors displayed in Figure 2.

### Plant growth, transformation, genotyping, and selection of homozygous lines

The binary plant transformation vectors (displayed in Figure 2) were utilized to transform Arabidopsis (Colombia-0) using the Agrobacterium-mediated (strain GV3101) floral dip method (Clough and Bent, 1998). Transformed T1-plants were selected on soil by spraying with 100 mg L^−1^ Basta herbicide (Bayer), resulting in a very clear distinguishable resistant/susceptible phenotype. DNA extracted via Invisorb Spin Plant Mini Kit (Invitek) from these hemizygous T0 plants was verified to contain the T-DNA fragment via PCR amplification. The T2 seeds harvested from the positive lines were sown on half-strength Murashige and Skoog medium supplemented with 10 g L^−1^ sucrose and 10 mg L^−1^ Basta (Bayer; Basta-selection medium). A total of approximately 200 seedlings were assigned to be resistant or susceptible, allowing confirmation of the expected 3:1 segregation ratio (chi-squared *P* > 0.05), enabling selection of single-locus transformation events. Approximately 40 T3 seeds, collected from these single-locus T2 Basta-resistant plants (10 for each event), were sown on Basta-selection medium, allowing retrieval of homozygous lines (1:0 segregation). All plants were grown in a 16/8 h light/dark regime at 21°C, 50% relative humidity, and 150 μmol m^−2^ s^−1^ white light. Seedlings were grown on half-strength Murashige and Skoog supplemented with 10 g L^−1^ sucrose for 15 d (harvested 15 d after germination). Tissue samples for both RNA extraction and metabolite quantification (see below) were harvested from the same in vitro plate. Multiple in vitro Murashige and Skoog-plates represent the different biological replicates.

### RNA extraction, expression, and statistical analysis

RNA was extracted from 50 mg of 15-d-old complete seedlings grown on half-strength Murashige and Skoog medium supplemented with 10 g L^−1^ sucrose. Plant material (seedlings) was flash-frozen and stored at −80°C until RNA extraction. RNA was extracted using GeneJET Plant RNA Purification Kit (ThermoFisher Scientific) using the manufacturer’s protocol. Potential contamination of DNA was averted via RNA-free DNAse treatment, using Ribolock RNAse inhibitor to limit RNA breakdown (extended ThermoFischer Scientific RNA purification protocol). A Bio-Rad iScript cDNA synthesis kit (containing oligo dTs and random primers) was used to convert 1 µg of RNA to cDNA. Subsequently, RT-qPCR was conducted on these cDNA samples using RT-qPCR primers shown in Supplemental Table S1. Schematic representation of the primer sets used to determine total and transgenic expression as well as the primer set used to analyze the sum of *TPK1* and *TPK2* expression is provided in Figure S2. qPCRBIO SyGreen Mix with Fluorescein (Sopachem) was utilized in the reaction mix. A RT-qPCR run comprised an initial denaturation at 95°C for 3 min and 40 cycles of denaturation at 95°C for 5 s, annealing for 20 s (variable temperature; Supplemental Table S1), and extension at 60°C for 6 s. Data analysis and normalization were performed using the qBASE software based on the 2^–ΔΔ^Ct method (Livak and Schmittgen, 2001; Hellemans et al., 2007). Normalization was obtained by amplification of *AtACTIN2* (At3g18780), *protein phosphatase 2A subunit A2 (PP2AA2;* At3g25800) and *RGS1-HXK1 interacting protein 1 (RHIP1;* At4g26410) as reference genes (Dekkers et al., 2012).

Statistical analysis was performed to examine the differences in mean expression as well as metabolite levels as compared to the WT. First, analysis of variance was performed to assess whether sample sets contained equal variance (*F*-test, Microsoft Excel). If no significant difference in sample variance was concluded (*P* > 0.05), sets were considered homoscedastic (if *P* < 0.05, sets were concluded to be heteroscedastic). Second, Student’s *t* test was performed to allow comparison of means (two-tailed, scedasticity depending on *F*-test outcome). Values were considered significant (*P < 0.05) or very significant (**P < 0.01).

### Quantification of metabolites via LC–MS/MS

Quantification of the different metabolites (HMP, total HMP, HET, total HET, thiamin, TMP, and TPP) was performed according to a recently developed LC–MS/MS method (Verstraete et al., 2020). This validated methodology allows adequate quantification of all aforementioned metabolites from a single Arabidopsis tissue sample. The metabolite determination utilized 120 mg of 15-d-old full seedling material (unless stated otherwise), grown on half-strength Murashige and Skoog medium supplemented with 10 g L^−1^ sucrose. Plant material (seedlings) was flash-frozen and stored at –80°C until the time of analysis. Each biological replicate was measured via two independent measurements (technical replicates, analyzed independently). The analytes were extracted from the samples during a 30 min heating step at 74°C. Subsequently, to allow quantification of both nonphosphorylated and phosphorylated metabolites (e.g. HET and HET-P), samples were split in two. To the first half, 10 U of acid phosphatase were added, followed by incubation at 45°C for 24 h. To the second half, the same volume of water was added, followed by incubation at 4°C for 24 h. This enabled the quantification of total-HET, total-HMP, and total B1 in the phosphatase-treated samples, whereas HET, HMP, thiamin, TMP, and TPP levels could be measured in the nonphosphatase-treated samples. Consequently, the presence of phosphorylated entities, such as HET-P and HMP-P(P), was indirectly measured by comparison of metabolite levels between phosphatase-treated and -untreated samples.

### Accession numbers

Accession numbers for the genes used in this study can be found in Supplemental Table S1.

## Supplemental data

The following materials are available in the online version of this article.

**Supplemental Figure S1**. Analysis of nonphosphorylated HMP and HET metabolite levels in transgenic Arabidopsis seedlings as analyzed by LC–MS/MS.

**Supplemental Figure S2**. Schematic representation of several primers used in RT-qPCR reactions.

**Supplemental Figure S3**. Relative abundance of metabolites in the WT and engineered Arabidopsis lines.

**Supplemental Figure S4**. Correlation between engineered gene expression and metabolite levels of the corresponding intermediate in THIC- and THI1-engineered lines.

**Supplemental Figure S5**. Phenotype of all soil-grown engineered lines.

**Supplemental Figure S6**. Analysis of metabolite levels in a selection of soil-grown Arabidopsis plants.

**Supplemental Figure S7**. Estimation of thiamin biosynthesis gene transcript levels in WT Arabidopsis.

**Supplemental Table 1**. Information regarding the different primer pairs.

## Supplementary Material

kiab198_Supplementary_Data

## Notes

D.V.D.S. and S.S. designed the experiments. S.S. conducted molecular cloning, plant transformation, genotypic analysis, expression analysis, phenotyping, and sampling of plant material. Molecular data analysis was done by S.S. and D.V.D.S. J.V. operated LC–MS/MS and analyzed the LC–MS/MS data together with C.S. S.S., and D.V.D.S wrote the manuscript. J.V. and C.S. commented and approved the manuscript. D.V.D.S. conceived and coordinated the project.

The author responsible for distribution of materials integral to the findings presented in this article in accordance with the policy described in the Instructions for Authors (https://academic.oup.com/plphys/pages/general-instructions) is: Dominique Van Der Straeten (Dominique.VanDerStraeten@UGent.be).
