# Supplementary material for: Metabolic engineering provides insight into the regulation of thiamin biosynthesis in plants
Source: Plant Physiol. 2021 May 4;186(4):1832–47. doi: 10.1093/plphys/kiab198 (PMC8331165; doi:10.1093/plphys/kiab198)
Supplement: kiab198_Supplementary_Data [file kiab198_supplementary_data.pdf]

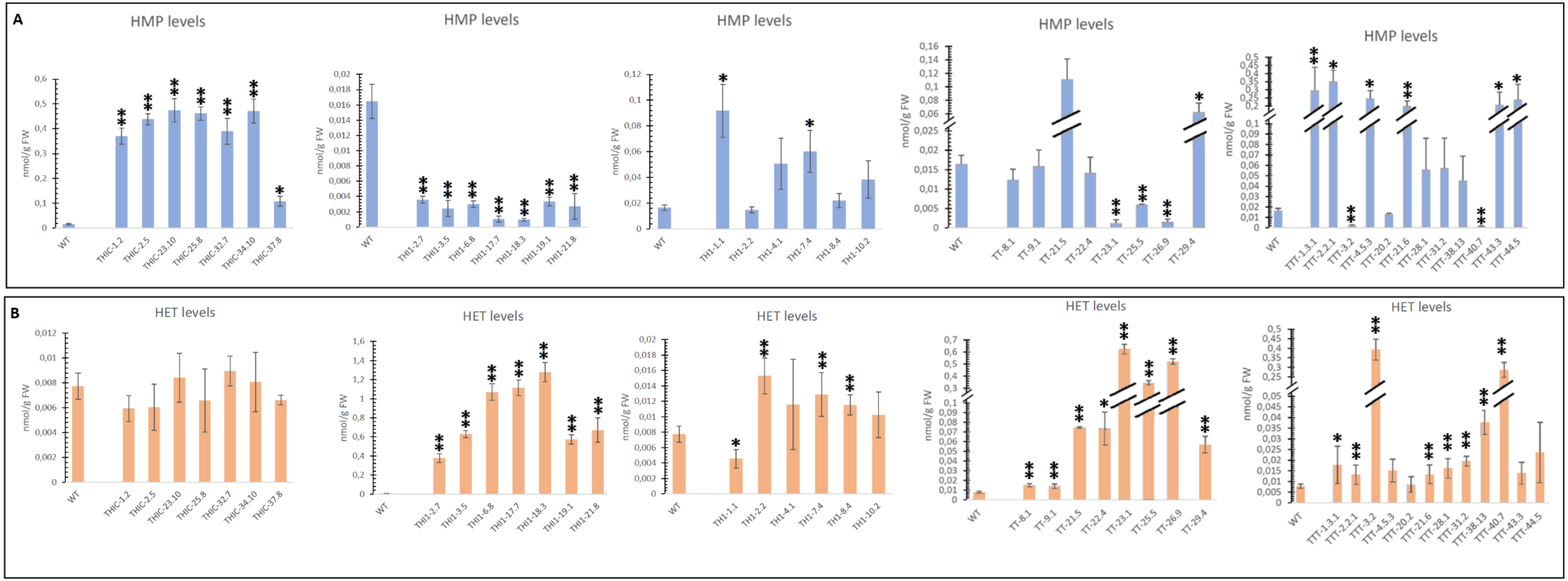

Supplemental figure S1. Analysis of non-phosphorylated HMP and HET metabolite levels in transgenic Arabidopsis seedlings as analyzed by LC-MS/MS. HMP **(A)** and HET **(B)** levels in single gene (*THIC*, *THI1*, and *TH1-lines*) and multigene-engineered (TT and TTT-lines) transgenic Arabidopsis lines are displayed. Analyses were performed on homozygous T3-lines, originating from independent plant transformation events. Lines TTT-1.3.1, TTT-2.2.1 and TTT-4.5.3 were analyzed in T4 generation (indicated by their 3-digit identifiers). Values are means (error bars indicate 95% confidence interval) of three independent biological repeats (WT: N=11). Data were obtained from 15-day old seedlings grown on half-strength Murashige and Skoog medium supplemented with 10g/liter sucrose. Samples were collected in the middle of the light period. Statistical difference compared to WT is indicated to be significant (single asterisk, $p < 0.05$) or very significant (double asterisks, $p < 0.01$). Quantification of non-phosphorylated metabolites was performed according to a validated LC-MS/MS method (Verstraete et al., 2020).

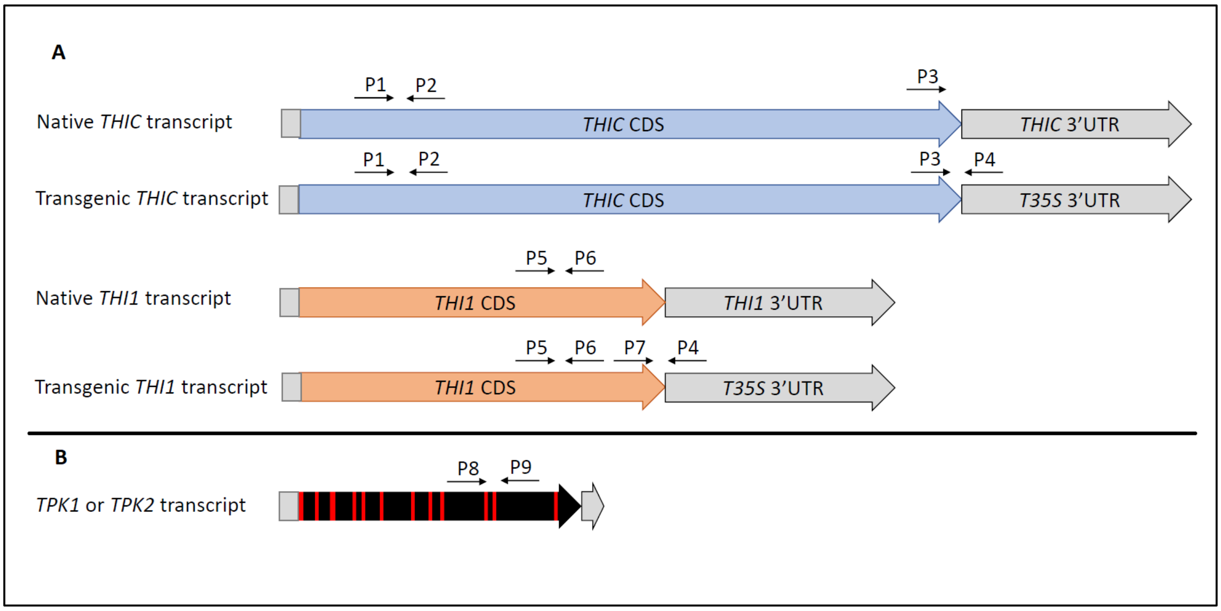

Supplemental figure S2. Schematic representation of several primers used in reverse transcription quantitative PCR reactions. 3'UTR's are indicated with gray arrows, while 5'UTR regions are indicated with a small gray block. Coding sequences (CDS) are represented by colored arrows (length of the arrow is relative to CDS length). **A** Different primer pairs were designed to investigate transcript levels of both native and transgenic transcript abundance of *THIC* and *THI1* in quantitative real-time PCR (qRT-PCR) analysis. Primers inside the CDS of the genes allowed quantification of both native and transgenic transcript levels. This is accomplished by pairs P1-P2 and P5-P6 for *THIC* and *THI1* expression, respectively. Unless specifically stated (as 'transgenic' expression), this total expression, meaning the sum of both native and transgenic expression is intended. Utilization of primers spanning the CDS-3'UTR border, which discriminates between native and transgenic transcripts, allows insight in the abundance of transgenic transcript upon their utilization in qRT-PCR analysis. This is accomplished by utilization of primer pairs P3-P4 and P7-P4 to allow insights in accumulation of transgenic *THIC* and *THI1* transcripts, respectively. **B** Designing of a primer pair in a specific region inside the CDS conserved among *TPK1* and *TPK2*, allows amplification of an (almost) identical transcript (equal length) and thereby allows quantification of total *TPK* transcript abundance (sum of *TPK1* and *TPK2*). Black zones inside the CDS represent conserved regions, while red zones indicate minute differences between *TPK1* and *TPK2*. **Gene information.** *THIC*, At2g29630, 1935 bp; *THI1*, At5g54770, 1050 bp; *TPK1*, At1g02880, 804 bp, *TPK2*, At2g44750, 804 bp.

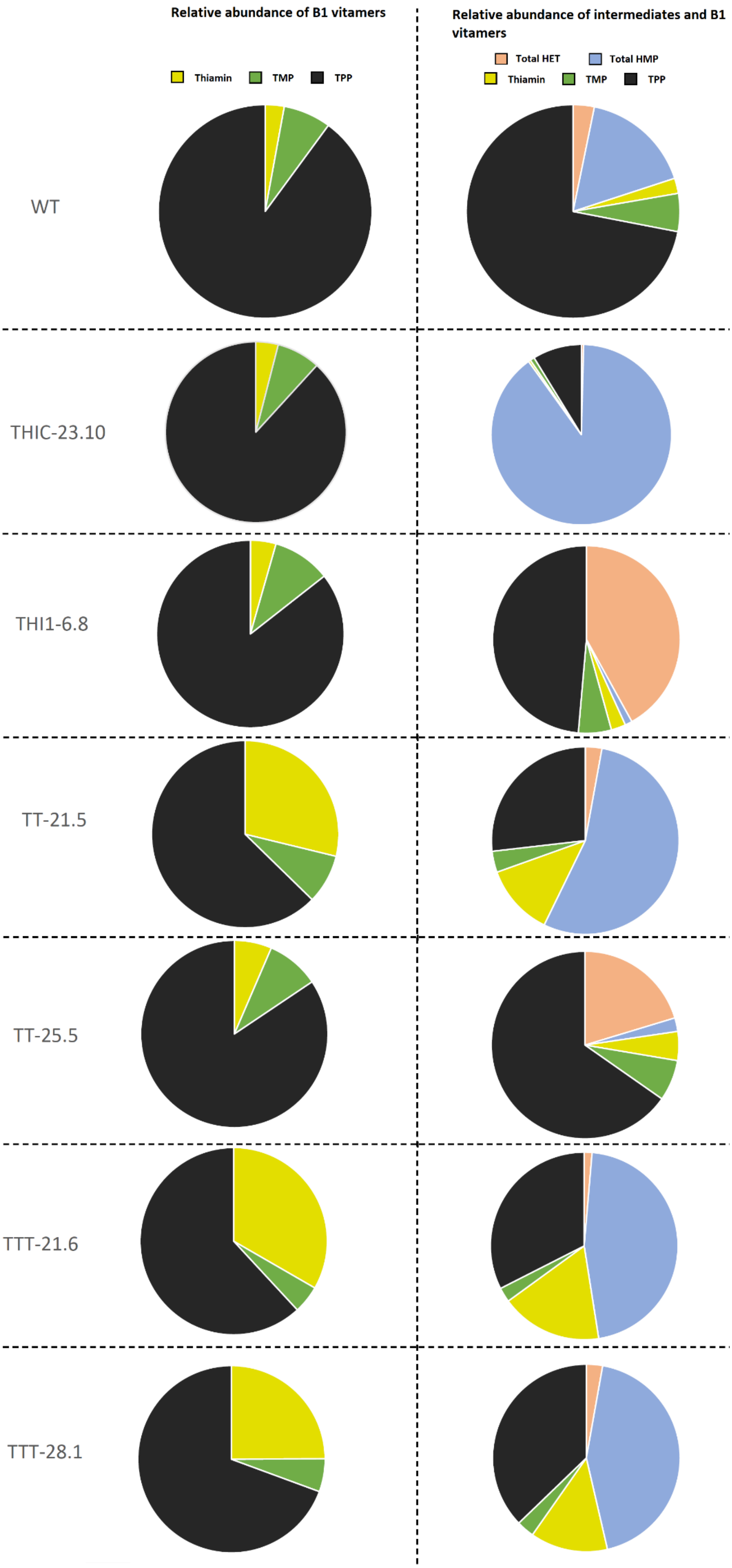
Relative abundance of B1 vitamers
Thiamin
TMP
TPP
Relative abundance of intermediates and B1 vitamers
Total HET
Total HMP
Thiamin
TMP
TPP
WT
THIC-23.10
THI1-6.8
TT-21.5
TT-25.5
TTT-21.6
TTT-28.1

Supplemental figure S3. Relative abundance of metabolites in the WT and engineered Arabidopsis lines. Left, pie-charts representing the relative molar abundance of different B1 vitamers (thiamin, TMP and TPP) in WT and transgenic lines. Right, pie-charts representing the relative molar abundance of metabolites committed towards B1 biosynthesis (total HMP, total HET, thiamin, TMP and TPP).

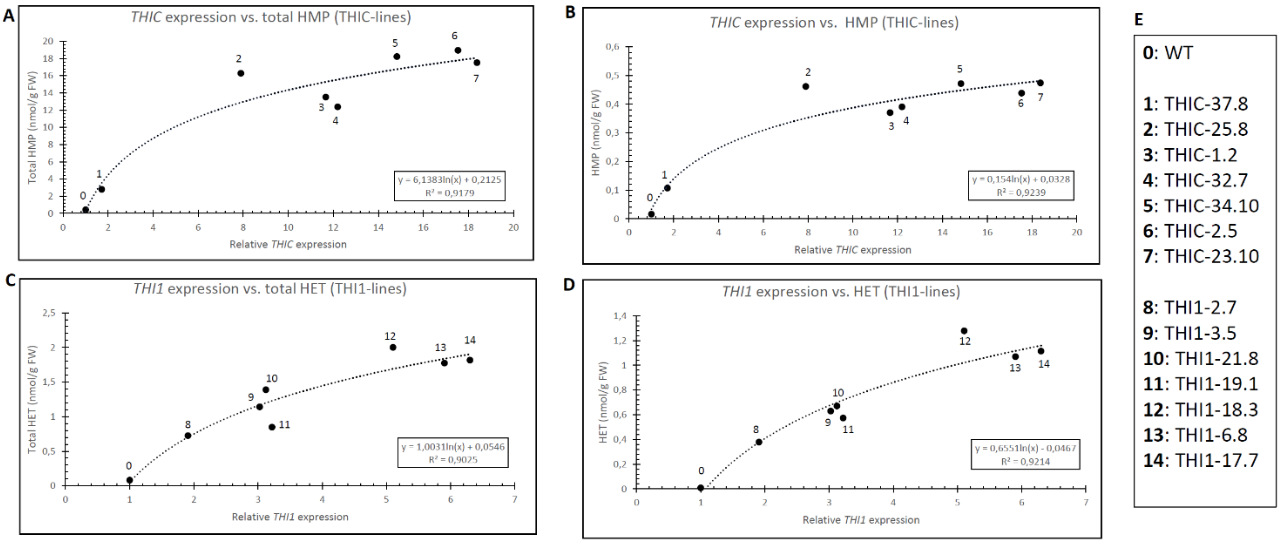

Supplemental figure S4. Correlation between engineered gene expression and metabolite levels of the corresponding intermediate in THIC- and THI1-engineered lines. In THIC-engineered lines, *THIC* expression correlated with accumulation of total HMP (**A**) as well as non-phosphorylated HMP (**B**). In THI1-engineered lines, *THI1* expression correlated with accumulation of total HET (**C**) as well as non-phosphorylated HET (**D**). The specific engineered lines depicted in the graphs are given a number code (**E**). Data points represent values for both metabolite content and expression levels, as displayed in Fig 3A and 3B. Logarithmic approximation of expression/metabolite prediction as well as correlation is displayed as calculated using Microsoft Excel. **Interpretation**. As the supply of the thiazole intermediate is potentially limiting B1 accumulation, there is a need to ameliorate metabolic engineering approaches to boost production of this metabolite. The high molar quantities of pyrimidine produced upon *THIC* overexpression, together with the potential toxicity of accumulating pyrimidine metabolites, indicates the need to balance *THIC* expression with thiazole production. This could be achieved by increasing both *THIC* and *THI1* expression to a level sufficient to provide an additional 2 nmol/g supply of pyrimidine and thiazole intermediates, respectively. Based on the observed correlation between gene expression and the accumulation of the corresponding intermediates, one can calculate that such levels could theoretically be reached with a 2-fold increase in *THIC* expression combined with 7-fold increase in *THI1* expression. The observation that the enhancement in thiamin achieved in the most successful lines (e.g. TTT-2.2.1, TTT-44.5) (see Fig. 4B), is in the same order of magnitude as the increment in the thiazole intermediate in THI1-engineered lines (up to 2 nmol/g FW, see Fig. 3B) further emphasizes the rate-limiting role to thiazole production.

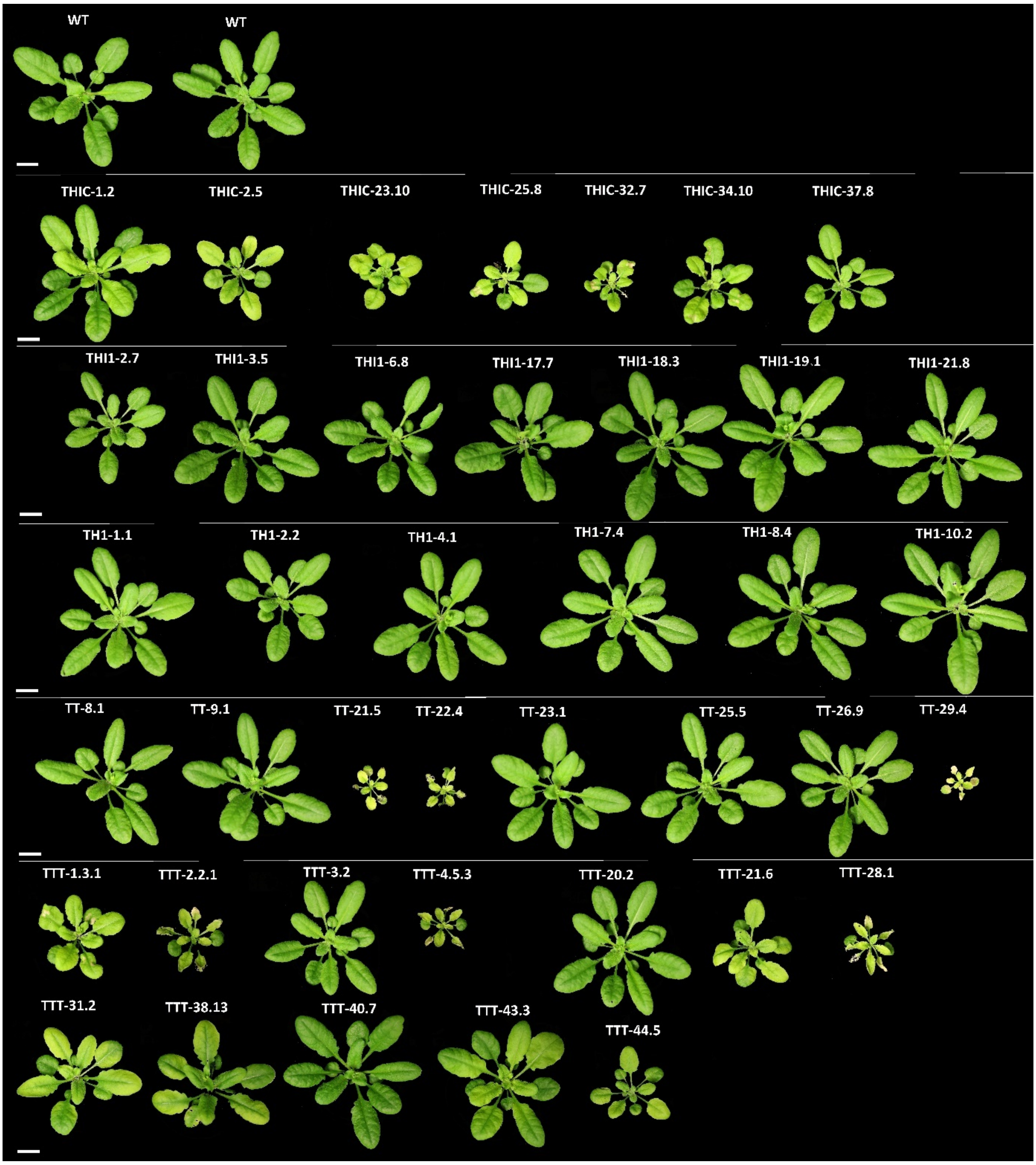

Supplemental figure S5. Phenotype of all soil-grown engineered lines. All lines were grown on soil (16/8 light/dark regime; 21 °C; 50% relative humidity; 150µmol m^{-2} S^{-1} white light) and photographed at 30 days of age. Rosettes were digitally extracted to allow comparison. White bars indicate 1 cm, all images including different lines, are set to the same scale. Designation of the line is indicated above the depicted plant. An aberrant phenotype was witnessed in *THIC* overexpressing lines, with the exception of line THIC-37.8, which depicted modest *THIC* overexpression (THI1 and TH1-engineered lines resembled WT). The typical phenotype included chlorosis and exaggerated leaf serrations.

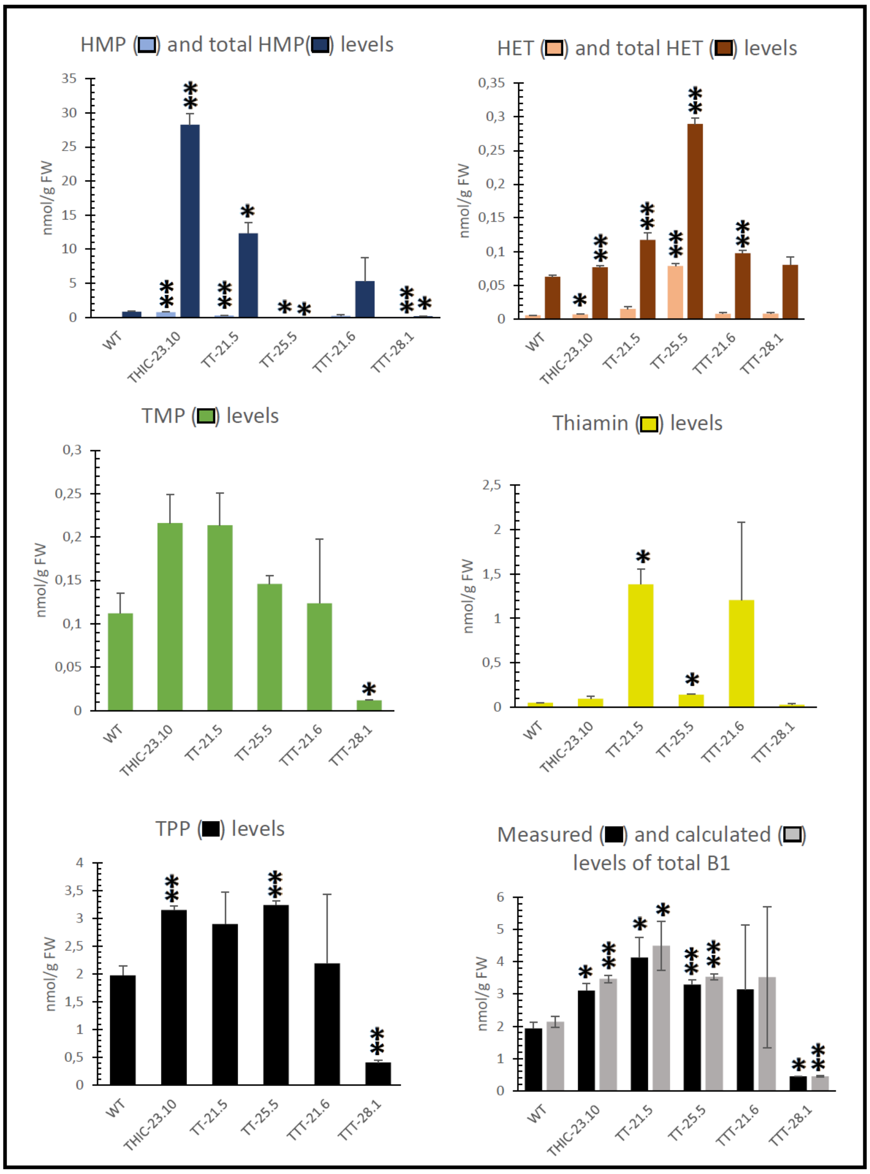

Supplemental figure S6. Analysis of metabolite levels in a selection of soil-grown Arabidopsis plants. Metabolite levels were measured via LC-MS/MS in Arabidopsis plants grown on soil for 30 days (Fig. S5). Samples are mean (±SE) of 3 biological repeats, each consisting of complete leaf tissues from multiple individual plants. Statistical difference compared to WT is indicated to be significant (single asterisk, $p < 0.05$) or very significant (double asterisks, $p < 0.01$).

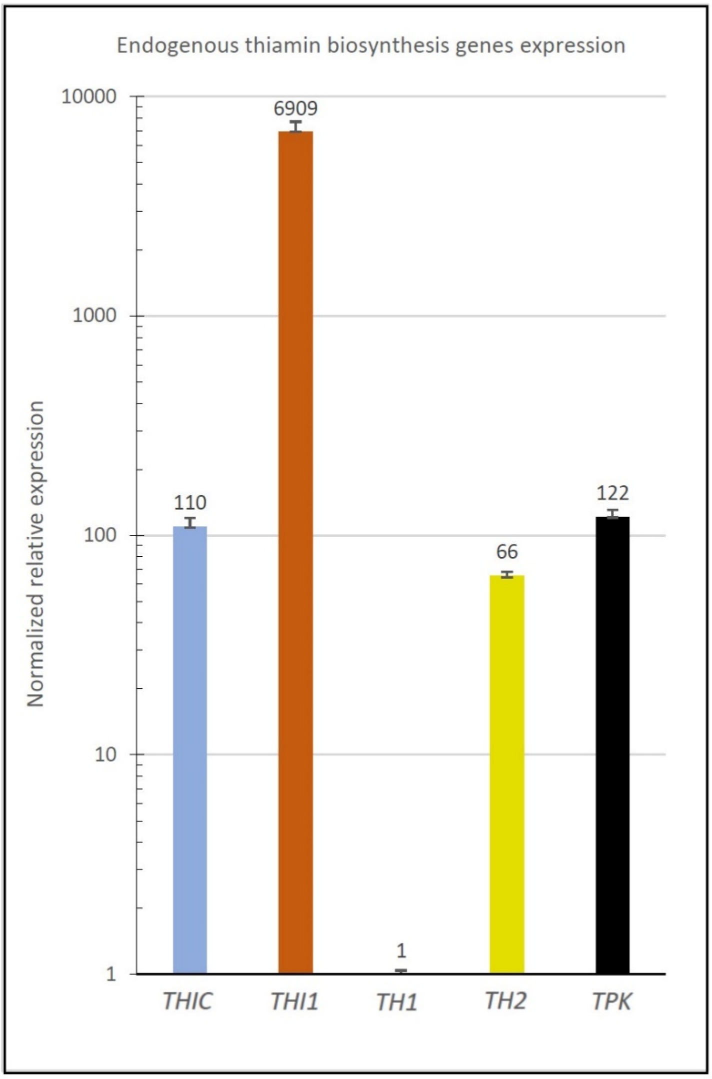

<u>Supplemental figure S7. Estimation of thiamin biosynthesis gene transcript levels in WT *Arabidopsis*.</u> Relative (endogenous) gene expression of thiamin biosynthesis genes *THIC*, *THI1*, *TH1, TH2* and *TPK1/TPK2* in WT is depicted, as revealed by real-time quantitative PCR (qRT-PCR). Values are semi-quantitative, as they are based on qRT-PCR threshold (Ct) values using different primer sets (cT values: *THIC*, 22.5; *THI1*, 17; *TH1*, 29.5; *TH2*, 23.5; *TPK*, 22.5). As all primer pairs are optimized to have adequate efficiency (95-105%), comparison of their raw Ct values, allows relative estimation of transcript abundance. Values for '*TPK*' comprise combined expression of *TPK1* and *TPK2* (primers target an amplicon in conserved region). Relative transcript levels of thiamin biosynthesis genes are depicted in which *TH1* expression is set to be 1 (values are relative to *TH1*). Data was collected from 15-day old seedlings grown on half-strength Murashige and Skoog medium supplemented with 10g/liter sucrose. Samples were collected in the middle of the light period. Values are means of three independent biological repeats (± standard error). Data analysis and normalization were performed using the qBASE software, based on the $2^{-\Delta\Delta Ct}$ method (Livak and Schmittgen, 2001; Hellemans et al., 2007) Specific information regarding primers and conditions can be retrieved from supplemental table S1.

Supplemental table 1. Information regarding the different primer pairs. Gateway cloning attB sequences are indicated in bold and italics. Gene specific parts in gateway cloning are underlined. Primer numbers of primers depicted in Fig. S2 are indicated in bold.

Gene	Locus	Forward primer (5'—3')	Reverse primer (5'—3')	Primer concentration	Annealing temperature	Function
THIC	At2g29630	GAACACAAGGAAGTTGTCCATGAAG **(P1)**	CCTTAGCTTAGCAAGCCCAATG **(P2)**	400 nM	60°C	qRT-PCR quantification of (*Arabidopsis*) *THIC* expression
THI1	At5g54770	CGCTATTGTGAGGTTGACCAGA **(P5)**	CAAAAGTTGGTCCCATTCTCG **(P6)**	400 nM	60°C	qRT-PCR quantification of (*Arabidopsis*) *THI1* expression (Bocobza et al., 2013)
TH1	At1g22940	GAACTCCGTTCTCCTCGCAT	CGACAAAGCGTTTAGCCACC	400 nM	60°C	qRT-PCR quantification of (*Arabidopsis*) *TH1* expression
TH2	At5g32470	GGGGTCTCATTTGTGCCTTTG	CTCCCATCCAAGAGCGAATG	400 nM	60°C	qRT-PCR quantification of (*Arabidopsis*) *TH2* expression (Hsieh et al., 2017)
TPK1/TPK2	At1g02880 / At2g44750	CCAACTCCTTCCAAAGACTCATCG **(P8)**	CCAGAGAAGATCCGAATCCGATTC **(P9)**	400 nM	60°C	qRT-PCR quantification of transgenic (*Arabidopsis*) combined *TPK1* and *TPK2* expression. Primers were designed in conserved region, equal size amplified products arise from *TPK1* and *TPK2* transcripts.
Trans THIC	/	catccgaaagtatgcagaggag **(P3)**	ccactttgtacaagaaagctggg **(P4)**	400 nM	60°C	qRT-PCR quantification of transgenic (*Arabidopsis*) *THIC*. Primers are designed to amplify a PCR product on the junction of CDS-3'UTR, thereby conferring specificity to the transgene.
Trans THI1	/	gttgttcctggtatgattgttacc **(P7)**	ccactttgtacaagaaagctggg **(P4)**	400 nM	60°C	qRT-PCR quantification of transgenic (*Arabidopsis*) *THI1*. Primers are designed to amplify a PCR product on the junction of CDS-3'UTR, thereby conferring specificity to the transgene.
PP2AA2	At3g25800	CAATGGTTACAAGACAAG	ATTCGGTAGAGATAGTGA	400 nM	58°C	qRT-PCR quantification normalization (*Arabidopsis* reference gene)
RHIP1	At4g26410	GGTCCGACATACCCATGATCC	GAGCTGAAGTGGCTTCCATGAC0	400 nM	58°C	qRT-PCR quantification normalization (*Arabidopsis* reference gene)
AtACTIN2	At3g18780	TCCACGAGACAAATAACTCAATCA	TGCAAGTGCTGTGATTTCTTTGCTCAT	400 nM	58°C	qRT-PCR quantification normalization (*Arabidopsis* reference gene)
THIC	At2g29630	***AAAAAGCAGGCTCAGCT***<u>ATGGCTGCTTCAGTACACTG</u>	***AGAAAGCTGGG***T<u>TTATTTCTGAGCAGCTTTGACATA</u>	400 nM	58°C	Cloning *Arabidopsis THIC*, primers contain partial attB sites for Gateway cloning
/	/	***GGGGACAAGTTTGTACAAAAAAGCAGGCT***	***GGGGACCACTTTGTACAAGAAAGCTGGGT***	400 nM	55°C	Cloning, full length attB fragment
THI1	At5g54770	***GGGGACAAGTTTGTACAAAAAAGCAGGCTTT***<u>ATGGCTGCCATAGCTTCTACTC</u>	***GGGGACCACTTTGTACAAGAAAGCTGGGT***<u>TTAAGCATCTACGGTTTCAGCTG</u>	400 nM	55°C	Cloning *Arabidopsis THI1*, primers contain attB sites for Gateway cloning

TH1	At1g22940	***GGGGACAAGTTTGTACAAAAAAGCAGGCTTA***ATGAATAGCTTAGGAGGAATTAGGAG	***GGGGACCACTTTGTACAAGAAAGCTGGGT***TCAAATTCCCCTTTTGCTC	400 nM	55°C	Cloning *Arabidopsis TH1*, primers contain attB sites for Gateway cloning

Supplemental references

HSIEH, W. Y., LIAO, J. C., WANG, H. T., HUNG, T. H., TSENG, C. C., CHUNG, T. Y. & HSIEH, M. H. 2017. The Arabidopsis thiamin-deficient mutant pale green1 lacks thiamin monophosphate phosphatase of the vitamin B-1 biosynthesis pathway. *Plant Journal,* 91**,** 145-157.

VERSTRAETE, J., STROBBE, S., VAN DER STRAETEN, D. & STOVE, C. 2020. The first comprehensive LC–MS/MS method allowing dissection of the thiamine pathway in plants. *Analytical Chemistry,* 92**,** 4073-4081.
